# Linking functional and structural dendritic spine remodeling during fear learning and extinction in vivo

**DOI:** 10.1126/sciadv.aec3961

**Published:** 2026-07-17

**Authors:** Xiaoyang Li, Qiyu Zheng, Kim Hoi Kin Wong, Kenneth Kin Yip Wong, Cora Sau Wan Lai

**Affiliations:** ^1^ School of Biomedical Sciences, LKS Faculty of Medicine, The University of Hong Kong, Hong Kong, China.; ^2^ Advanced Biomedical Instrumentation Centre, Hong Kong Science Park, Shatin, New Territories, Hong Kong, China.; ^3^ Department of Electrical and Electronic Engineering, The University of Hong Kong, Hong Kong, China.

## Abstract

Structural plasticity of dendritic spines has been observed during different learning paradigms, but how the functional dynamics of dendritic spines changes with memory processing and how these patterns relate to structural plasticity and dendritic integration remain unclear. Here, we perform longitudinal functional and structural in vivo imaging of the frontal association cortex in mice subject to fear conditioning and extinction over several days. We show that fear learning induced responsive spines that are more likely to be synchronous and clustered, which are consolidated over the following days but attenuated by extinction. We develop a causal inference model demonstrating that the active spine calcium signals during fear learning prevent the spines from being eliminated while promoting the elimination of neighboring spines after memory consolidation. Furthermore, the dendritic responsive signal reveals a learning-dependent tone discrimination pattern that is correlated to spines’ structural remodeling. Our findings provide in vivo evidence consistent with functional-structural link of dendritic spines in a bidirectional learning paradigm.

## Teaser

Fear learning and extinction bidirectionally modulate dendritic spines’ remodeling that contributes to dendritic processing.

## INTRODUCTION

Synapses serve as fundamental units for neuronal communication and information processing in the mammalian brain (*1*–*4*). The dynamic functional and structural changes of synapses after activity-dependent potentiation and depotentiation of postsynaptic dendritic spines are crucial for learning and encoding memory engrams (*5*–*11*). The plasticity of dendritic spines enables neuronal circuit to encode new experience, such as learning, or adaptively update previously acquired information, e.g., extinction (*12*–*14*). Previous studies have highlighted the substantial dendritic spine structural remodeling in associative learning and unlearning (*14*–*16*). For example, in the mouse frontal association cortex (FrA) that integrates multimodal sensory inputs (*17*), fear conditioning (FC) induces elimination of dendritic spines, whereas subsequent extinction promotes the formation of new spines at positions closely adjacent to previously eliminated spines (*12*). This reversible and spatially precise structural remodeling suggests a finely tuned mechanism by which the neural circuit dynamically updates associative memories through selective elimination or reformation of synaptic connections.

The spatial and temporal organizations of dendritic spines have been demonstrated to facilitate dendritic information processing through nonlinear mechanisms of calcium integration (*2*, *4*, *18*–*20*). The clustered plasticity has been demonstrated by numerous in vitro and ex vivo studies investigating how combinations of presynaptic inputs trigger dendritic potentiation (*2*, *21*–*24*). However, establishing direct links between these mechanisms and the experience-driven rewiring of synaptic connectivity networks remain challenging. Recent seminal studies using in vivo calcium and structural imaging on layer (L) 2/3 and L5 basal and apical dendritic spines or axonal boutons in the primary motor cortex of mice during motor task training have advanced our understanding (*25*–*29*). These studies have shown that motor learning–induced spine formation preferentially occurs in task-biased dendrites (*25*). The calcium activities of newly formed spines synchronize with their dendrites, and dendritic Ca^2+^ spikes occurring during rapid eye movement sleep play a crucial role in the elimination and strengthening of new spines for motor learning (*26*). In addition, the functional coactivity of dendritic spines during motor learning or decision-making was compartmentalized and clustered within dendrites (*27*, *28*). These findings provide valuable insights into how learning experiences directly shape dendritic computation through targeted synaptic reorganization. Nevertheless, how bidirectional behavioral conditions, such as fear learning and extinction, modulate the functional and structural dynamics of dendritic spines in relation to dendritic function is yet to be explored in vivo. Such behavioral paradigms would particularly be informative for uncovering mechanisms underlying the remodeling of dendritic spine.

In this study, we performed in vivo longitudinal calcium and structural imaging on apical dendritic spines of L5 pyramidal neurons (PNs) in the FrA of mice undergoing FC and subsequent fear extinction. We chose FrA for the study on the basis of the previous studies showing that the FrA integrates auditory cues and amygdala inputs for auditory cues during FC and PNs’ activation in the FrA, and its upstream inputs contribute to memory formation in the associative fear learning paradigm (*30*, *31*). Our data reveal complex relationships between dendritic spine calcium signals and physical modifications in response to conditioned stimuli, including structural plasticity and spatially clustering patterns. We further validate the importance of functional-structural interactions in associative fear learning via the computational neural network. These functional-structural interactions contribute significantly to the overall dendritic response and tone discrimination, both of which are shaped by fear learning and extinction experiences. Our findings link the coordinated functional and structural plasticity of dendritic spines that correlate to dendritic functional dynamics in fear learning and extinction.

## RESULTS

### Fear learning–modulated CS response in the overall spine population

**Fig. 1.**
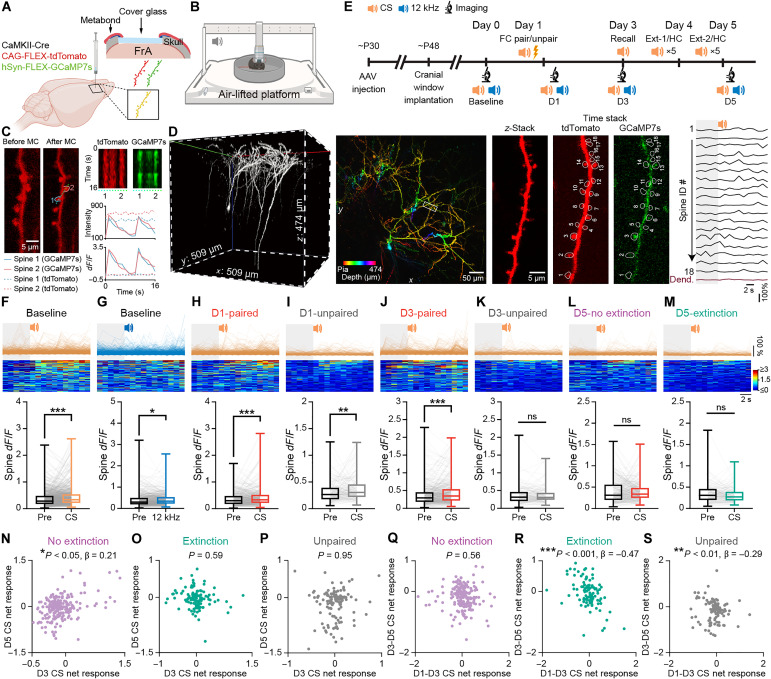
Dendritic spine calcium dynamics in response to FC and fear extinction. (**A**) Schematic of virus injection and cranial window implantation on the FrA. (**B**) Head-fixed mouse on an air-lifted platform during two-photon imaging [created in BioRender. C. S. W. Lai (2026), https://biorender.com/y51u182]. (**C**) Representative dendritic spine images before and after motion correction (MC). Left: Maximum projection of tdTomato. Right top: Kymograph of the dash line drawn in the left image. Right bottom: Raw intensity and *dF*/*F* of spines shown in the left image. (**D**) Left two: Representative 3D reconstruction and pseudocolored *z*-stack map of PN dendrites. Right: Zoomed region (white box in the *z*-stack map) and the calcium signals of corresponding spines in the baseline session. (**E**) Experimental timeline (see Materials and Methods). Ext, extinction; HC, home cage. (**F** to **M**) Top: Spine calcium traces in response to CS or 12-kHz tone. Gray shadings show the pre-CS period. Middle: Heatmaps show calcium dynamics of 100 dendritic spines with the largest CS net response. Bottom: Mean spine calcium *dF*/*F* during the pretone period (Pre) versus tone period (CS or 12-kHz tone) of all imaged dendritic spines. Data are shown as box-whisker plots; each gray line represents the activity change of one spine. *n =* 623 spines [(F) and (G)]; *n =* 454 (H); *n =* 157 (I); *n =* 430 (J); *n =* 144 (K); *n =* 275 spines (L); *n =* 143 (M); *P < 0.05, **P < 0.01, and ***P < 0.001; ns, no significant; linear mixed-effects model. (**N** to **S**) Linear mixed-effects model with FDR correction testing the correlation of spine CS net response or net response change between two different imaging sessions with estimated regression coefficient (β). *n =* 273 spines (N); *n =* 141 (O); *n =* 136 (P); *n =* 216 (Q); *n =* 117 (R); *n =* 119 (S).

To investigate how structural plasticity and calcium dynamics of dendritic spines change in response to FC and fear extinction, we used in vivo two-photon microscopy to image the dendritic spines in the FrA of mice. We delivered a set of adeno-associated viruses (AAVs) to the FrA for sparse colabeling of L5 PNs expressing tdTomato and GCaMP7s, enabling simultaneous imaging of spine structures and calcium signals of the apical dendrites in head-fixed mice allowed to locomote on an air-lifted platform (Fig. 1, A to C). To maintain stable fluorescence levels across multiple-day imaging, we simultaneously acquired GCaMP7s signals and dendritic spine structure (tdTomato) at 1 Hz (fig. S1). After the motion correction, we show that the GCaMP7s signal was not affected by structural imaging of tdTomato (Fig. 1C). Before the serial imaging sessions with behavioral experiments, a three-dimensional (3D) structural map identifying different cortical layer PNs was acquired to select dendrites exclusively from L5 PNs. These selected dendrites were relatively isolated from the other compartments on the same plane to avoid the artifacts from out-of-focus structures (Fig. 1D). We manually registered the region of interest (ROI) of dendritic spines on the basis of structural imaging, and the calcium signals of each ROI were acquired after motion correction. To measure the baseline response, we examined the calcium signals of spines in response to the future conditioned stimulus (CS; 4-kHz tone) and a control stimulus (12-kHz tone) (Fig. 1E).

Before the FC, both stimuli elicited significant increases in the overall dendritic spine calcium signal (Fig. 1, F and G). Mice were subsequently subjected to either FC-paired (coterminated CS and shock) or unpaired control (not coterminated, random CS and shock) at day 1 (D1), with imaging session performed immediately (within 1 hour) after FC and at day 3 (D3) (Fig. 1E and fig. S2, G to I). In the D1 imaging session, the overall spine calcium signal significantly increased in both paired and unpaired groups in response to CS (Fig. 1, H and I). Notably, no significant responses to the 12-kHz tone were observed in either group (fig. S2A), indicating that fear learning leads to an immediate CS preference of the overall dendritic spine responsiveness. In the D3 imaging session (2 days after conditioning), the overall spine CS responsiveness persisted in the paired group, with spines exhibiting significantly higher calcium signal to CS and decreased signal to the 12-kHz tone compared to pretone levels (Fig. 1J and fig. S2A). In contrast, no significant difference was observed between pretone signal and the tone-induced response to CS or to 12-kHz tone in the unpaired group at D3 (Fig. 1K and fig. S2A), suggesting that the spine CS responsiveness is consolidated after fear learning in the paired group but not in the unpaired group. Next, we further investigated the effects of fear extinction on the dendritic spine’s calcium dynamics. Mice in the paired group were further divided into two groups: (i) extinction group, which underwent repeated exposures of CSs over two consecutive days, and (ii) no-extinction group, which stayed in the home cage (Fig. 1E). The imaging session was conducted at day 5 (D5) to examine the calcium signals of dendritic spines after fear extinction. Neither the extinction group nor the no-extinction group exhibited a significant increase in the overall spine calcium signal in response to CS (Fig. 1, L and M). However, we observed that dendritic spines exhibited a significant and positive correlation between the CS net response at D3 and D5 in the no-extinction group (Fig. 1N). Conversely, this positive correlation between the spine CS net response at D3 and that at D5 was not observed in the extinction or unpaired group (Fig. 1, O and P). Moreover, spines that had increased CS net response from D1 to D3 showed diminished CS net response after fear extinction (Fig. 1, Q to S), indicating that FC-induced increment of CS net response in selective spine population was reversed by extinction. The CS response of the overall spine population did not show a significant change at D5 (Fig. 1, K to M), suggesting that long-term memory consolidation may rely on sparse coding, in which only a subset of neurons or synapses contributes.

To better illustrate the dynamics of CS responses across the overall spine population, we also examined longitudinal changes within each subgroup (fig. S2B). Although the initial CS response in the unpaired group was different from the other groups at the baseline, the unpaired group exhibited a significant CS response at D1, which subsequently decreased by D3 (fig. S2B). Nevertheless, we acknowledge that the overall spine calcium response may not be sufficiently informative on its own; thus, analyses focusing on spine subpopulations defined by structural plasticity and dendritic features were examined in the following sections.

### Clustered spine synchronicity contributes to dendritic integration

**Fig. 2.**
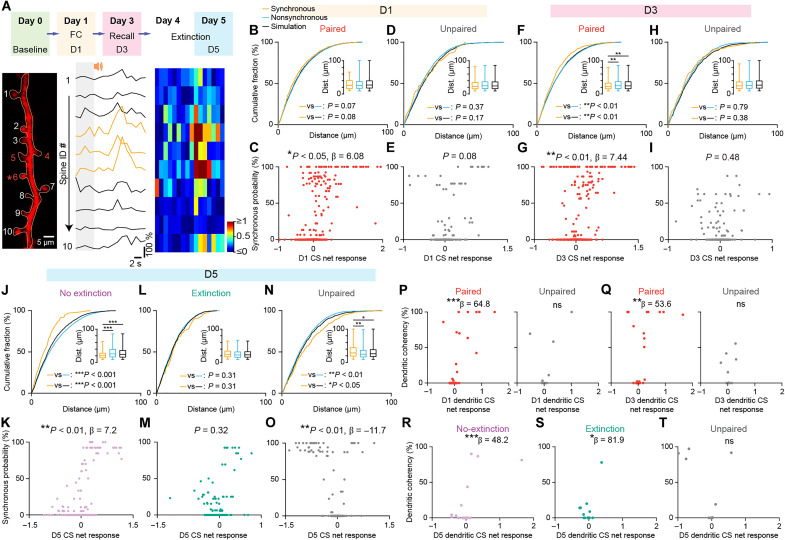
Dendritic spine CS responses correlate with spine synchronicity on dendrites. (**A**) Top: Experimental timeline of imaging sessions. Bottom left: Representative dendrite segment showing multiple dendritic spines. The red color labels the synchronous spines to the red-starred spine #6. Bottom middle and right: Activity traces and heatmaps of the dendritic spines corresponding to the left dendrite segment. (**B**, **D**, **F**, **H**, **J**, **L**, and **N**) Cumulative distribution of the intrapair distance (insets: Box-whisker plots of interpair distance). A two-sample Kolmogorov-Smirnov test with FDR correction was used. *n =* 451 synchronous pairs and *n =* 6289 nonsynchronous pairs (B); *n =* 135 and *n =* 2046 (D); *n =* 489 and *n =* 6271 (F); *n =* 93 and *n =* 2088 (H); *n =* 204 and *n =* 4743 (J); *n =* 123 and *n =* 1670 (L); *n =* 250 and *n =* 1931 (N). The random simulation was performed for 1000 times. (**C**, **E**, **G**, **I**, **K**, **M**, and **O**) Linear mixed-effects model testing the correlation of the spine synchronous probability and spine CS net response with the estimated regression coefficient (β). *n =* 454 spines (C); *n =* 157 (E); *n =* 430 (G); *n =* 144 spines (I); *n =* 275 (K); *n =* 143 (M); *n =* 137 (O). (**P** to **T**) Linear mixed-effects model testing the correlation of the dendritic CS net response and dendritic coherency at D1 (P), D3 (Q), and D5 [(R) to (T)]. Red dots represent the paired group, gray dots represent the unpaired group, purple dots represent the no-extinction group, and green dots represent the extinction group. *n =* 37 dendrites [(P), paired]; *n =* 12 [(P), unpaired]; *n =* 37 [(Q), paired]; *n =* 12 [(Q), unpaired]; *n =* 23 (R); *n =* 14 (S); *n =* 12 (T).

To assess the functional coordination of dendritic spines within a dendrite, we analyzed the pairwise temporal correlation of calcium activities of dendritic spines (*28*, *32*). We classified spine pairs with positively correlated activities [multiple correlation analysis with false discovery rate (FDR) correction] as synchronous pairs and the rest as nonsynchronous pairs (Fig. 2A). In the paired fear-conditioning group, we first found that synchronous spine pairs tended to be more closely spaced than nonsynchronous pairs, although this effect did not reach significance at D1 (Fig. 2B; synchronous versus nonsynchronous, *P* = 0.07). In addition, the synchronous probability of each spine (defined as the proportion of synchronous spines on the same dendrite; see Materials and Methods) was significantly and positively correlated with that spine’s CS net response (Fig. 2C). These relationships were not observed in the unpaired group (Fig. 2, D and E). On D3, we observed a significantly closer proximity of synchronous pairs exclusively in the paired group (Fig. 2, F and H). Spines exhibiting a higher CS net response at D3 were more likely to be synchronous with their counterparts only in the paired group (Fig. 2, G and I). Our data indicate that associative fear learning promoted the synchronous activity of CS responsive spines. The proximity of synchronous spines and the positive correlation between CS net response and spine synchronous probability persisted at D5 (Fig. 2, J and K), with a shorter distance between synchronous spines over time in the no-extinction group (fig. S3, D, G, and J), suggesting a further consolidation of this spatial-temporal pattern. However, these correlations were disrupted in the extinction group (Fig. 2, L and M, and fig. S3, E, H, and J). Notably, instances in which a spine’s synchronous probability approached 100% may reflect dendrite-wide calcium transients driven by backpropagating action potentials (bAPs) (*25*, *28*, *33*, *34*). After excluding spine pairs likely dominated by such dendrite-wide events, we still observed a significant positive relationship between spine synchronous probability and CS net response (fig. S3, A to C), suggesting that the synchronicity effects reported here are unlikely to be explained solely by bAP-related signals. The coactivity phenomenon of clustered dendritic spines has also been reported in motor learning, in different phases of decision-making and in response to visual stimuli (*27*, *28*, *32*, *35*). Our findings further suggest that the spatiotemporal interplay of the dendritic spine calcium dynamics carries information related to fear memory that could be strengthened under consolidation or attenuated by extinction.

Previous studies have shown that cooperation of dendritic spines with temporal coactivity promotes nonlinear computation of dendrites by amplifying and enabling local processing of information on dendritic segments (*36*–*38*). To clarify how the temporally synchronous spine pairs contribute to the dendritic information processing, we analyzed the relationship between the dendritic shaft CS net response and the dendritic coherency (defined as the proportion of synchronous spine pairs on the corresponding dendrite; see Materials and Methods). We found that dendrites with higher dendritic coherency exhibited increased dendritic CS net response in the paired group during the D1, D3, and D5 imaging sessions (Fig. 2, P to R). A positive correlation between the dendritic shaft CS net response and the dendritic coherency was also observed in the extinction group (Fig. 2S), even though shaft CS net responses in this group were predominantly negative (fig. S3K). By contrast, no such relationship was detected in the unpaired group at any session (Fig. 2, P, Q, and T). Together, these results support a tight relationship between dendritic spine synchronicity (dendritic coherency) and dendritic shaft activity that might be modulated by fear learning.

**Fig. 3.**
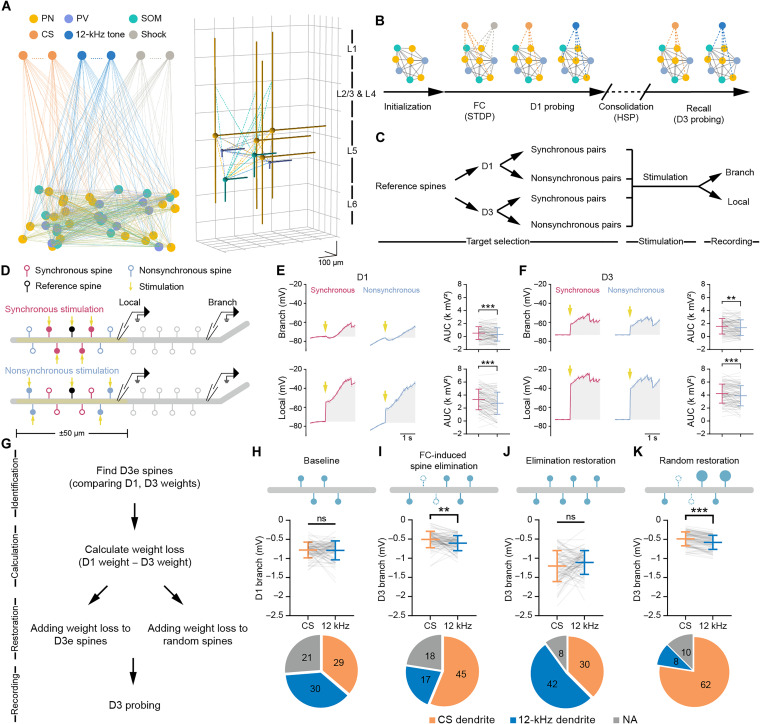
Validation of functional synchronicity and structural elimination of dendritic spines in a computational neural network model. (**A**) Left: Schematic diagram of neuronal populations for the computational neural network including PN, PV, SOM, “CS” stimulation neuron, “12-kHz tone” stimulation neuron, and “shock” neuron. Right: Representative neuronal connections in the cortical region of the neural network model. (**B**) Timeline of FC simulation in the computational neural network model. (**C**) Experimental design for examining effects of synchronous spine stimulation on dendritic signal integration. (**D**) The schematic diagram shows the simultaneous stimulation of the reference spine with its synchronous or nonsynchronous pairs and the recording of the local dendritic segment or whole dendritic branch. (**E** and **F**) Left: Curves (means ± SEM) show the dendritic potential change before and after artificial stimulation. The shadowing area denotes the area under the curve (AUC) of the change of dendritic potential after stimulation. Right: Quantification of the AUC upon stimulation of synchronous spines or nonsynchronous spines. **P < 0.01 and ***P < 0.001, paired *t* test; sample size in selected dendrite: *n =* 100. Data are shown as the means ± SD. (**G**) Experimental design for examining effects of spine elimination on the dendritic CS response. (**H** to **K**) Top: Schematics show the spine plasticity from the baseline (H) to D3 after consolidation (I) and after the restoration of weight to eliminated spines (J) or to random spines (K). Middle: Quantification of dendritic potential toward CS or 12-kHz tone stimulation during probing. **P < 0.01 and ***P < 0.001, paired *t* test; sample size in dendrite: *n =* 80. Bottom: Pie charts show the number of dendritic branches that exhibit a significantly higher response to CS than to 12-kHz tone, that to 12-kHz tone than to CS, and no preference (NA). Data are shown as the means ± SD.

To investigate how synaptic synchronicity affects dendritic computation, we developed a biophysically detailed neural network model on the basis of the NEURON simulator (*39*, *40*). This computational approach circumvents the technical challenges of selectively measuring dendritic outputs driven by synchronous versus nonsynchronous spine activation. Our model incorporates excitatory PNs, inhibitory parvalbumin interneurons (PVs), somatostatin interneurons (SOMs), and long-projecting neurons responding to diverse stimuli, i.e., CS, 12-kHz tone, and shock (Fig. 3A; see Materials and Methods). We trained the neural network model with a simulation of associative fear learning (Fig. 3B) (*41*). We then randomly selected reference spines in PNs and identified their corresponding synchronous and nonsynchronous spines for the further artificial stimulation experiments (Fig. 3C). Similar to the experimental observations, synchronous spines were located more proximal to the reference spines than nonsynchronous spines in the neural network model (fig. S3L). By artificially stimulating the reference spines simultaneously with their synchronous or nonsynchronous spine population (Fig. 3D), we found that stimulating synchronous spine population elicited a significantly stronger dendritic response than stimulating nonsynchronous spines (Fig. 3, E and F). This effect was consistently observed in both D1 and D3 probing and was evident in both local dendritic segments and across the entire dendritic branch. To assess the robustness of the simulations, we performed sensitivity analyses by varying key model parameters, including the spike timing–dependent plasticity (STDP) time constant, the magnitude of STDP weight updates, and the homeostatic synaptic plasticity (HSP) time constant. Across these parameter settings, the model consistently supported the same conclusion: Synchronous spine costimulation promotes dendritic activation (table S2). Thus, our computational finding, which is consistent with experimental observations, indicates that synaptic synchronicity modulated by fear learning and consolidation is patterned and contributing to dendritic signal integration.

### Dendritic spine elimination fate is inferred from its own and its neighboring spines’ calcium signals to CS

In addition to the functional plasticity of dendritic spine, associative fear learning has been shown to influence the structural plasticity of dendritic spines, a crucial process for cognitive functions (*7*, *13*, *42*). Our previous findings demonstrated that FC induced the elimination of L5 PN apical dendritic spines in the FrA, correlating with freezing behavior (*12*, *43*). Despite the recent advance in transient optical techniques to erase structural long-term potentiation in vivo (*44*), it remains challenging to selectively prevent spine elimination, making it difficult to directly assess the role of spine elimination in learning animals (*45*). To investigate how the spine elimination affects the CS-evoked dendritic signal integration, we thus took leverage on the neural network model and performed artificial restoration of weight loss on the learning-induced eliminated spines (Fig. 3G). In our simulation, each spine is represented by a synaptic weight with a bounded range (0 to 15). We defined D3-eliminated spines (D3e spines) as those that were present before associative learning (weight >3) but became absent by the recall session (weight <3). By examining the dendritic potentiation in response to stimuli by administering CS or 12-kHz tone stimulations (probing), we first observed that fear learning led to significantly greater dendritic response toward CS than toward 12-kHz tone (Fig. 3, H and I). After the weight restoration for eliminated spines, the dendritic CS response was lowered, and the number of dendrites exhibiting a stronger response to CS than to 12-kHz tone decreased (Fig. 3J). We further confirmed this effect with a range of model parameters (table S2). In the random restoration control that added weight loss to random spines of the corresponding dendrite, the increased dendritic CS response remained (Fig. 3K). These results suggest that learning-induced spine elimination plays a vital role in dendritic integration to CS.

**Fig. 4.**
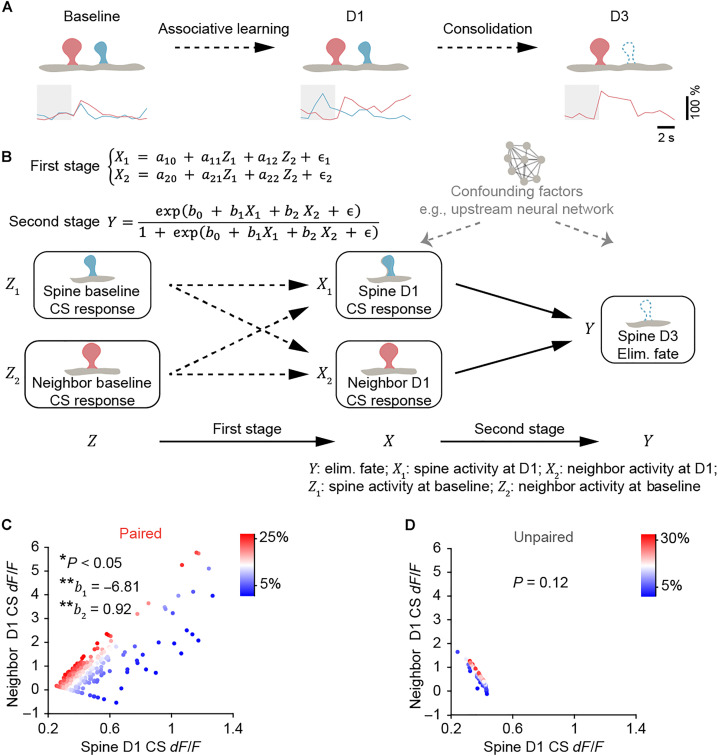
Causal inference of spine elimination with a two-stage least squares model. (**A**) Schematic diagrams show the structural changes of two representative neighboring dendritic spines (<5 μm) (top) and their calcium traces in response to CS during the baseline, D1, and D3 (bottom). (**B**) Graphical representation of the two-stage least squares model. First stage: Linear regression of variable *X* (CS response of the target spine and its neighboring spines in FC) and instrumental variable *Z* (CS response of the target spine and its neighboring spines in baseline). Second stage: Logistic regression of dependent variable *Y* (elimination fate of the target spine) and the regressed prediction from the first stage. The two-stage least squares model uses the instrumental variable to eliminate the influences of confounding factors. (**C** and **D**) Scatterplot of the elimination probability predicted by the two-stage least squares model (represented by the color bar) and the CS *dF*/*F* of the target spine and its neighboring spines in FC. The two-stage least squares model was performed as illustrated in (B). For (C), the first-stage coefficients *a*_11_ = 0.22, *a*_12_ = 0.11, *a*_21_ = −0.51, and *a*_22_ = 1.08; *n =* 376 spines. For (D), *n =* 128 spines.

Given the importance of spine elimination, we next sought to unravel its underlying mechanisms by investigating the factors determining dendritic spine elimination during FC. To address this question, we analyzed the calcium signals of the FC-eliminated dendritic spines and their neighboring spines within a 5-μm radius (Fig. 4A). We first performed a simple correlation analysis between the calcium signal of the individual spine or those of neighboring spines at D1 and their elimination status from D1 to D3. We found that spine’s elimination probability negatively correlated with its calcium signal at D1 (fig. S4A), but this correlation was not observed when analyzed with the neighboring spines (fig. S4B).

While these observational correlations provide valuable insights, deducing the causal relationships leading to spine elimination requires more sophisticated causal inference methods (*46*–*48*). The core challenge in the causal inference arises from confounding factors (*49*). In our case, unobserved confounders, such as emergent upstream neural network states, could potentially influence both variable *X* (the signal of target spine or neighboring spines) and variable *Y* (whether the spine would be eliminated after consolidation) (Fig. 4B), complicating the interpretation of the interaction between the spine’s signal during fear learning and its elimination fate. To adjust for potential confounding effects, we used an instrumental variable approach, which has been widely applied in various research fields from economics and genomics to neuroscience (*48*, *50*, *51*). We identified an instrumental variable *Z* that causally infers the independent variable of interest *X* but only affects the dependent variable *Y* through *X* (Fig. 4B). We posited that the dendritic spine calcium signal in the baseline represented a neutral and randomized state in a stable neural network. Thus, the baseline calcium level of the target spine or neighboring spines, defined as variable *Z*, would affect the spine elimination (variable *Y*) exclusively through its effect on the spine calcium signal in the D1 imaging session (variable *X*), thereby excluding the confounding factors. We constructed a predictive model of the independent variable *X* (*X*_1_, signal of the target spine at D1, and *X*_2_, signal of neighboring spines at D1) on the basis of the instrument *Z* (*Z*_1_, signal of the target spine in the baseline, and *Z*_2_, signal of neighboring spines in the baseline) (the first-stage regression). We then applied this prediction in the second-stage regression to estimate the causal effects of *X*_1_ and *X*_2_ on the dependent variable *Y* (spine elimination fate). In the paired group after fear learning, the spine CS response in situ negatively influenced its chance of elimination, whereas the signal of its neighboring spines showed a positive impact on its elimination (Fig. 4C). In particular, despite no direct correlation observed between the spine elimination and the neighboring spines’ signal (fig. S4B), the causal inference, which corrected the measurements from unmeasured confounding factors, helped indicate the hidden competitive impact of spine calcium signals on the structural elimination of neighboring spines (*52*). No significant result was observed for the two-stage least squares regression in the unpaired group (Fig. 4D). This conclusion remained consistent when we varied the spatial radius used to define neighboring spines (fig. S4, C to E). Our data suggest that the higher CS response of the individual spine protects itself against the fate of elimination, while the CS response of its neighboring spines instead promotes the chance of elimination. These findings imply a competitive relationship between neighboring spines in terms of memory consolidation, providing insights into the mechanisms underlying fear learning–induced dendritic spine elimination in the FrA.

### Different structurally plastic spine populations exhibit functional uniqueness

**Fig. 5.**
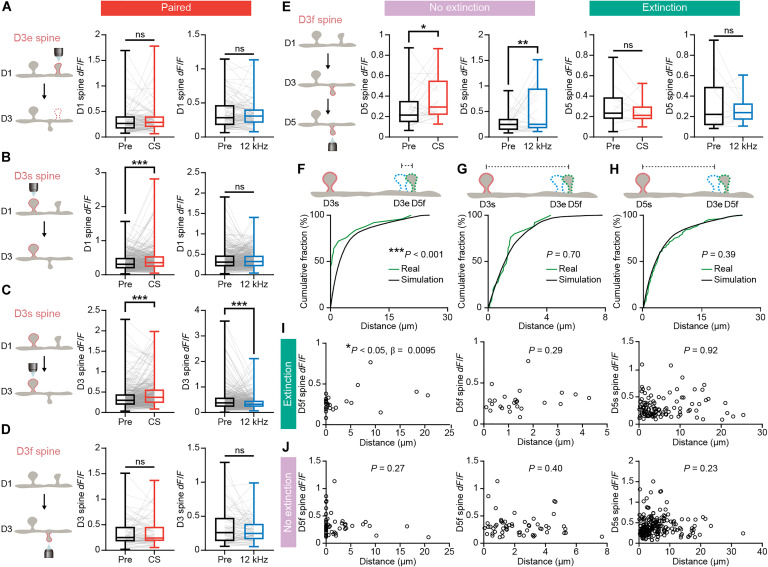
Functional and spatial properties of dendritic spines under diverse structural plasticity. (**A** to **E**) Quantification of the mean calcium *dF*/*F* during the pretone period (Pre) versus tone period (CS or 12-kHz tone) of D3e spines in the D1 imaging session (A), D3s spines at D1 (B), D3s spines at D3 (C), D3f spines at D3 (D), and D3f at D5 (E). *P < 0.05, **P < 0.01, and ***P < 0.001; ns, not significant; linear mixed-effects model; *n =* 105 spines (A); *n =* 349 (B); *n =* 349 (C); *n =* 81 (D); *n =* 25 [(E), no extinction]; *n =* 12 [(E), extinction]. Data are shown as box-whisker plots. (**F** to **H**) Cumulative distribution function of the closest distance between D5f spines and D3e spines [(F), *n =* 16], between D5f spines and D3s spines [(G), *n =* 26], and between D5s spines and D3e spines [(H), *n =* 108]. The simulation was performed 1000 times on the basis of the random distribution of D3e (F and H) or D5s (G). The two-sample Kolmogorov-Smirnov test was performed between real data and simulation data. (**I** and **J**) Linear mixed-effects model testing the correlation between the calcium *dF*/*F* of D5f spine and its closest distance to D3e spine [left, (I), *n =* 25 spines; (J), *n =* 58 spines], between the calcium *dF*/*F* of D5f spine and its closest distance to D3s spine [middle, (I), *n =* 25; (J), *n =* 58], and between the calcium *dF*/*F* of D5s spine and its closest distance to D3e spine [right, (I), *n =* 108; (J), *n =* 205].

With the finding that spine functional dynamics influence its elimination after fear memory consolidation, we next explored the functional uniqueness of spine populations on the basis of their fear learning–induced structural plasticity. Thus, we compared the calcium signals among different spine types according to the structural plasticity from D1 to D3, classified as D3-eliminated (D3e), D3-stable (D3s), and D3-formed (D3f) spines. Our analysis revealed that after FC in the D1 imaging session, D3e spines showed no significant differences in response to CS or 12-kHz tone compared to the pre-event period (Fig. 5A). In contrast, D3s spines exhibited CS responsiveness in both the paired and unpaired groups at D1 (Fig. 5B and fig. S5B). Tracking the calcium responses of D3s spines at D3, we found that D3s spines showed a CS-responsiveness preference only in the paired group, evidenced by the increased CS response and decreased calcium signal to the control 12-kHz tone (Fig. 5C), suggesting the consolidation of the CS-responsiveness property of D3s spines in paired fear learning. For D3f spines, although no significant response to CS was observed at D3, they became CS-responsive after an additional 2-day consolidation period in the D5 session in the no-extinction group (Fig. 5, D and E). Notably, extinction training disrupted the CS responsiveness of D3f spines at D5 (Fig. 5E).

Previous studies have demonstrated the remodeling of dendritic spine structural plasticity in close proximity within a dendrite in FC and fear extinction (*12*, *13*). To evaluate the relationship of newly formed spine localization and dendritic CS response in extinction, we measured the distances between D5-formed (D5f) and D3e spines (Fig. 5F), D5f and D3s spines (Fig. 5G), and D5-stable (D5s) and D3e spines (Fig. 5H), respectively, in the extinction group. We found that D5f spines were located significantly closer to D3e spines compared to randomly distributed simulation (Fig. 5F), suggesting that fear extinction–induced spine formation potentially rewired with the previous presynaptic connectivity. Furthermore, we observed a significant and positive correlation between the D5f spine CS response and their distances to D3e spines in the extinction group (Fig. 5I), indicating that the D5f spines located proximal to D3e spines were less CS-responsive. No such correlation was observed in the no-extinction group (Fig. 5J), suggesting that this spatial-functional association observed in D5f spines was induced by fear extinction. These data collectively demonstrate that dendritic spine structural plasticity reflects memory experience (consolidation or extinction) through corresponding changes in calcium signals and spatial distribution of dendritic spines.

### Spine remodeling contributes to tone discrimination on dendritic level

**Fig. 6.**
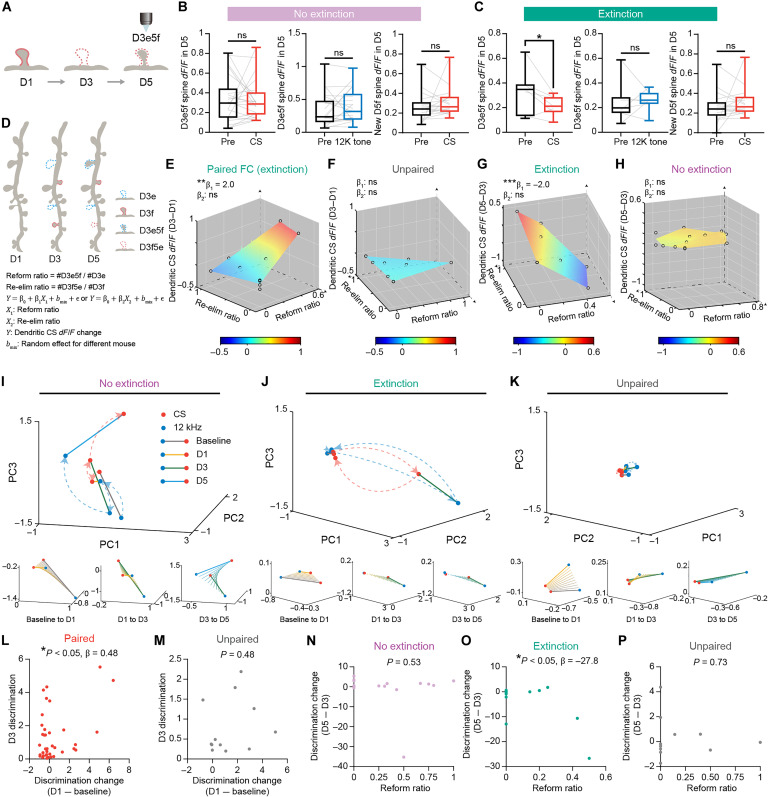
Structural remodeling of spines is associated with dendritic tone discrimination modulated by FC and fear extinction. (**A**) Definition of D3e5f spine. (**B** to **C**) Mean *dF*/*F* during the pretone period (Pre) versus tone-playing period [CS or 12-kHz (12K) tone] of D3e5f spines at D5. *P < 0.05; ns, not significant; linear mixed-effects model; *n =* 22 spines [(B), D3e5f]; *n* = 36 [(B), new D5f]; *n* = 7 [(C), D3e5f]; *n* = 18 [(C), new D5f]. Data are shown as box-whisker plots. (**D**) Schematic definition of dendritic reform and re-elim ratios and the equations of mixed-effects regressions [(E) to (H)]. (**E** to **H**) 3D scatterplot of dendrites in reform ratio, re-elim ratio, and shaft CS *dF*/*F*. β_1_ and β_2_ denote regression coefficients for reform and re-elim ratio. **P < 0.01 and ***P < 0.001; ns, not significant; *n =* 10 dendrites (E); *n =* 11 dendrites (F); *n =* 10 dendrites (G); *n =* 13 dendrites (H). Each circle represents one dendrite; the surface color indicates dendritic CS *dF*/*F*. (**I** to **K**) Top: Example PCA trajectories showing dendritic responsive patterns to CS (red dots) versus 12-kHz tone (blue dots) across different sessions. Line segments connecting red and blue dots show the tone discrimination at each session, and dashed arrows show the trajectories of discrimination changing from one session to another. Bottom: Zoomed transition trajectories. Color gradients reflect linear interpolation between sessions. (**L** and **M**) Linear mixed-effects model testing the correlation of the dendritic D3 discrimination index and discrimination change from D0 to D1; *n =* 37 dendrites (L); *n =* 12 (M). (**N** to **P**) Linear mixed-effects model testing the correlation of discrimination change from D3 to D5 and the reform ratio of the dendrite; *n =* 18 dendrites (N); *n =* 12 (O); *n =* 12 (P).

To further investigate the effects of opposite structural plasticity of dendritic spines on dendritic calcium activity in fear memory consolidation and extinction, we defined the reformed (D3e5f) spines as the dendritic spines formed at D5 located in close proximity to the position of the D3e spines (within 0.7 μm) and the re-eliminated (D3f5e) spines as the subgroup of D3f spines eliminated at D5 (Fig. 6A). We observed that reformed spines exhibited significantly decreased calcium signal to CS in the fear extinction group (Fig. 6C), whereas responses to the 12-kHz control tone and CS responses in new D5f spines were not reduced (Fig. 6C). In addition, there were no obvious calcium changes to CS or 12-kHz tone in the no-extinction or unpaired group (Fig. 6B and fig. S6A). Re-eliminated spines showed no significant difference in response to either CS or 12-kHz tone in any group (fig. S6C). Given that the calcium signal of extinction-induced newly formed spines decreased with the distance to the locations of D3e spines (Fig. 5I), the reduced CS response in reformed spines might suggest the location-specific information that counteracts CS responsiveness. However, it is noted that there were a relatively small number of animals contributing to the D3e5f spine analyses. To strengthen the interpretation and provide an internal control, we added an additional comparison using new D5f spines (spines that first appeared at D5 and were absent at prior time points; Fig. 6, B and C). The reduced CS-evoked calcium responses were specific to the reformed D3e5f spines after extinction, whereas responses to the 12-kHz control tone and CS responses in new D5f spines were not reduced (Fig. 6, B and C).

To assess the contribution of synaptic plasticity remodeling on dendritic computation, we first calculated the ratio of reformed spines (reform ratio) and re-eliminated spines (re-elim ratio) on dendrites (Fig. 6D). We then examined how the structural plasticity remodeling of spine affects the dendritic CS calcium response (Fig. 6D) by using linear mixed-effects regression to control the interanimal variation. We found that the reform ratio positively correlated with the increment in the dendritic CS response after FC (from D1 to D3) in the paired group (Fig. 6E). No significant correlation was observed between the reform ratio and the dendritic CS response change from D1 to D3 in the no-extinction or unpaired groups (Fig. 6F and fig. S7B). The reformed spines represent a subset of fear learning–induced eliminated spines that subsequently undergo structural restoration following extinction. Thus, our data highlight the role of the elimination of this selective spine population (reformed spines) in fear memory consolidation. Furthermore, at D5 after extinction, a higher reform ratio predicted a greater decrease in dendritic CS response (Fig. 6G). No correlations between the reform ratio and the dendritic CS response change were found in the no-extinction or unpaired group; or between the reform or re-elim ratio and the dendritic response change to 12-kHz tone across any of the groups (Fig. 6H and fig. S7). Together, these findings indicate the opposite effects of reformed spines on dendritic CS response during memory consolidation and extinction, confirming that the structural plasticity remodeling (i.e., reformation) of spines predicts the dendritic integration of fear memory.

FC induces distinct neuronal activation patterns in response to CS, which differ from the responses to control stimuli (*53*, *54*). However, the mechanisms underlying stimulus discrimination at the dendritic processing level remain poorly understood. To address this, we aligned calcium signal traces from all dendrites in every imaging session and performed principal component analysis (PCA) to capture the principal features contributing the most to the dendritic patterns (*55*, *56*). We calculated a discrimination index by measuring the Euclidean distance between the pairwise dendritic response features to CS and to 12-kHz tone in each session [line segments in Fig. 6 (I to K)], as well as the change of discrimination index (discrimination change) across sessions (fig. S8). This index represents how different is the dendrite in response to CS versus 12-kHz tone. We found no significant difference in discrimination change from the baseline to D1 between paired and unpaired groups after D1 FC (fig. S8B). However, after FC, there was a significant increase in the discrimination change from D1 to D3 in the paired group compared to the unpaired group (fig. S8C), suggesting the diverse dendritic responses to CS versus 12-kHz tone after consolidation of paired fear learning over 2 days. We also observed a positive correlation between the D1-baseline discrimination change (from the baseline to D1) and the D3 discrimination index in the paired group (Fig. 6L), indicating the selective consolidation of tone discrimination, but not in the unpaired group (Fig. 6M). Conversely, in the unpaired group, the larger increment in the discrimination change from the baseline to D1 corresponded to the stronger decrement in that during the memory consolidation period (from D1 to D3) (fig. S9), explaining the failure of memory consolidation in the unpaired group (Fig. 6M). Furthermore, the discrimination index decreased after fear extinction (fig. S8, A and D, and Fig. 6J), showing the reversibility of dendritic computation on tone discrimination under FC and fear extinction. Our data showed that the discrimination index is positively correlated with dendritic CS coherency at each imaging session (fig. S8E), indicating the link between spine synchronicity and dendritic tone discrimination. Our data suggest that fear learning and extinction not only affect the strength of the dendritic response but also modulate the dendritic discrimination pattern bidirectionally.

Given the critical role of dendritic spine structural remodeling in dendritic integration, we further investigated how the remodeling plasticity is associated with dendritic tone discrimination. We observed a positive correlation between the dendritic discrimination change from D1 to D3 and the reform ratio (fig. S10A), suggesting that the elimination of reformed spines might contribute to the distinguished dendritic response pattern to the conditioned tone in memory consolidation. However, after extinction, we found that an increase in the reform ratio corresponded to a decrease in the tone discrimination change (Fig. 6O). No significant correlations were found in the no-extinction or unpaired groups (Fig. 6, N and P). These findings suggest that the structural remodeling of learning-induced eliminated spines after extinction diminishes the learning-induced dendritic tone discrimination. Thus, the remodeling of dendritic spine plasticity (eliminated from D1 to D3 and reformed from D3 to D5) corresponds to the reversibility of dendritic calcium integration and tone discrimination computation, highlighting the role of dendritic spine structural remodeling in the information processing of the dendrite in fear learning and extinction.

## DISCUSSION

Our results provide fresh insights into the relationship between calcium dynamics and the structural plasticity of cortical dendritic spines during FC and fear extinction. Using longitudinal in vivo two-photon microscopy, we linked the functional properties of dendritic spines to their structural reorganization. Our findings demonstrated that the functional synchronicity among dendritic spines occurred in a physically clustered manner, influencing their structural plasticity in a heterosynaptic and competitive manner. These coordinated functional and structural dynamics contribute to shaping dendritic integration. Furthermore, we showed that the pattern of dendritic calcium dynamics represented tone discrimination, which is associated with dendritic spine remodeling under bidirectional learning and extinction paradigm. Our findings elucidate the relationship between the functional and structural plasticity of dendritic spines and their involvement in dendritic signal integration during associative learning in vivo.

The activity-dependent functional modifications of synapses and the associated structural plasticity of dendritic spines have been hypothesized as the substrates of memory (*3*, *9*, *25*). Previous in vitro and in vivo studies using uncaging of glutamate have yielded valuable insights about spine dynamics with different spatiotemporal multispine stimulations in acute experiments and shed light on the nonlinear integration of synaptic inputs at the dendritic level (*2*, *22*–*24*). Our study builds on and extends this work by directly linking the memory-associated functional and structural dynamics of dendritic spines across days in FC and fear extinction in vivo. We observed the physiological process of memory consolidation and revision with the emergence of functional and structural remodeling by opposite learning in FC and fear extinction. The FrA, located in the lateral region of the agranular cortex, receives inputs from the basolateral amygdala (BLA) and sensory cortices and sends projections to the medial prefrontal cortex (*12*, *17*, *57*). Prior work indicates that the FrA participates in stimulus integration required for associative memory formation, with FrA activity and upstream inputs—including perirhinal and insular cortices and the BLA—contributing to learning in associative paradigms (*30*). Causal circuit-level evidence further supports a role for BLA to FrA signaling in integrating auditory information, as perturbing this pathway during learning impairs auditory fear memory formation (*31*). Although other brain regions are also essential for FC (*58*–*60*), the FrA has been proposed to integrate a variety of sensory information that forms memory traces with distinct emotional values (*7*, *30*, *31*, *43*), and studies report robust and opposing spine remodeling in the FrA following FC and fear extinction (*12*, *43*) Together, these circuit- and system-level findings motivate focusing on the FrA as a locus for studying associative fear learning. Here, we extend this framework to the synaptic scale by showing the immediate and persistent auditory cue–evoked and learning-dependent responsiveness of dendritic spines and dendrites of L5 PNs in the FrA, illustrating the participation of the FrA in associative fear learning.

Prior work reports an analogous pattern of opposing structural remodeling in the auditory cortex, where FC increases spine formation and extinction preferentially eliminates the newly formed spines. In contrast, the FrA exhibits increased spine elimination after conditioning and spine reformation after extinction. Together, these observations—similar in logic but opposite in direction across cortical areas—support the view that learning-related synaptic remodeling is circuit- and region-specific rather than a single canonical signature of fear learning. With respect to how spine elimination could be associated with enhanced dendritic responsiveness, a naïve expectation is that fewer spines should reduce responsiveness. Instead, our results are consistent with selective remodeling that increases the signal-to-noise ratio: Elimination may preferentially remove weak, noisy, or poorly informative inputs, thereby increasing the relative impact of task-relevant synapses on dendritic responses. This interpretation is consistent with theoretical and experimental frameworks in which pruning can improve circuit efficiency and information transfer by removing suboptimal synaptic connections (*61*–*63*). Consistent with this view, in our data, spines that were later eliminated (D3e) showed little CS responsiveness at D1 (Fig. 5A), whereas spines that persisted (D3s) exhibited significant CS responsiveness at the same time point (Fig. 5B). Moreover, our simulations indicate that restoring weights at eliminated spine locations did not increase dendritic CS responses and instead produced a slight reduction (Fig. 3, I and J), while applying comparable weight increases to random spine locations had little effect on dendritic excitation (Fig. 3K), supporting the idea that dendritic responsiveness depends more on which specific synaptic connections are selectively retained and strengthened than on spine number per se. Growing evidence has demonstrated the cross-talk of stimulated and unstimulated spines within a dendritic segment leading to both homosynaptic and heterosynaptic plasticity (*64*, *65*). Recent studies using two-photon glutamate uncaging for multispine stimulation have demonstrated a competitive interaction between physically nearby spines in a calcineurin- and metabotropic glutamate receptor–dependent mechanism (*66*–*68*). The outcomes of dendritic structural modifications and the spreading range of heterosynaptic effects might be due to various factors, such as the spatial-temporal multispine stimulation protocol, dendritic spine density, inhibitory synaptic inputs on spines and dendritic shaft, and various properties of the dendrites (*65*, *66*, *68*, *69*). Here, using a natural learning paradigm in vivo, we develop a statistically causal inference model showing that the fear learning–specific CS calcium response of dendritic spine not only facilitates the spine’s survival during the memory consolidation process (homosynaptic) but also promotes neighboring spines’ elimination (heterosynaptic), likely due to the competitive mechanisms within the dendritic segment (*66*, *69*). This homeostatic regulation of synaptic weights has been proposed to be vital to constrain total synaptic weights within a stable physiological range for overall resource saving (*33*). The *N*-methyl-d-aspartate receptor activation of spines could spread calcium signals locally in dendrites and into neighboring spines, thereby promoting the spine coordination (*21*, *64*, *70*). We also observed spine synchronicity within an anatomically closer range after memory consolidation (∼11 μm; fig. S3G). Such proximity (10 to 20 μm) in spine coactivity was especially patterned on the apical but not basal dendrites in the motor cortex when training a mouse with motor learning (*27*) and also reported as compartmentalized and clustered within branches over ∼10 μm in different decision-making phases (*28*). This local synaptic coactivity may further trigger local dendritic depotentiation more strongly through the intracellular calcium release to promote learning and memory storage (*71*, *72*). Our findings associate this synaptic synchronicity to dendritic excitability with empirical and computational simulation evidence and further reveal that this local functional interaction is experience-relevant and modulated by fear learning and extinction (Fig. 2).

In our in vivo data, some of the synchronous probability of dendritic spines reaching 100% could potentially come from dendritic calcium transients that are associated with bAPs (*25*, *28*, *33*, *34*). By removing these potentially dendrite-driven synchronous pairs, the remaining ones show a positive correlation between spine synchronous probability and CS net response level at an even higher significance level (fig. S3, A to C), suggesting that our observations in spine synchronicity are unlikely to be solely contributed by the potential bAPs. Moreover, two additional features of our dataset argue against a purely global, branch-wide explanation for dendritic coherency: Synchronous spine pairs are spatially clustered (shorter interspine distances than expected; Fig. 2, F to I), which is less consistent with uniform global events that should drive most spines similarly across a branch, and extinction changes the organization of synchronous pairs rather than producing only a uniform scaling of activity (Fig. 2, J to M), suggesting learning-dependent reorganization of dendritic inputs rather than a simple change in overall excitability. A previous study by Kerlin *et al.* (*28*) simultaneously imaged calcium signals of dendritic spines, dendrites, and somata, demonstrating that linear regression–based bAP subtraction does not accurately unmix local spine calcium signals from bAPs in the motor cortex mice in a tactile decision-making task. They instead introduced an approach that deconvolved the reference signal in soma or apical trunk, in which they found that the proportion of spines related to animal behaviors was similar with or without bAP subtraction (*28*). Other studies also showed that the somatic bAP influence on calcium signals of apical dendrites is limited and, when present, it is difficult to isolate reliably (*34*, *73*).

The coordination of spatially organized spines reflects the resource-saving strategy of dendritic computation in long-term memory consolidation (*59*). Using in vivo two-photon imaging in *Thy1*-YFP (yellow fluorescent protein) mice or dual-eGRASP (enhanced green fluorescent protein reconstitution across synaptic partners) labeling, it has been demonstrated that fear extinction induces an opposite structural change that is previously induced by FC in either CS-specific or engram-specific manners, which can be further reversed again by fear reconditioning (*12*, *13*, *16*). The remodeling of structural plasticity induced by FC, fear extinction, and fear reconditioning supports the “unlearning” hypothesis of extinction (*71*, *72*) despite the fear renewal or fear reinstatement phenomena in the animal behaviors suggesting that fear extinction is a “new learning” instead (*59*, *73*). In the current study, by combining calcium and structural imaging of dendritic spines, we illustrate the opposite changes of spines in both structural plasticity and the functional dynamics that are highly relevant to and modulated by FC and fear extinction. The pruning of dendritic spines during fear learning was also accompanied by a selective strengthening process of CS responsiveness within a small fraction of stable spines and an increasing CS response of fear learning–induced newly formed spines over time (Figs. 1P and 5C). Together with the shorter intrapair distance of synchronous spine pairs over memory consolidation that was disrupted by extinction (fig. S3, D to J), these data show the overall recruitment and refinement of the new and existing circuits initiated by “unlearning,” echoing with the previous literature findings (*3*, *74*, *75*).

In addition to the “unlearning” nature of dendritic spine remodeling, we find that extinction-induced newly formed spines counteract the learning responses: These reformed spines exhibit lower CS calcium signals and negatively correlate with the overall dendritic response and stimulus discrimination on the dendritic level (Fig. 6, C, G, and O). The counteracting effects of reformed spines gained from memory extinction are not present before FC (Fig. 6C and fig. S6B). This counteraction might potentially encode for new information and contribute to increasing the capacity of information storage, thereby supporting the “new learning” hypothesis of extinction (*71*). While these findings point to distinct contributions of spine plasticity subclasses, these subtype-resolved interpretations should be viewed in the context of the limited number of learning-related spines represented in some plasticity categories in the current study.

Our findings indicate that the functional and structural remodeling of dendritic spines contributes to dendritic information processing both “unlearning” and “new learning” frameworks, thereby supporting behavioral flexibility in opposite associative learning. Yet, how specific memory traces allocated in different dendrites collaborate to support the memory computation of the neuron should be the subject of further work. In summary, we provide in vivo and longitudinal evidence linking the functional and structural dynamics of dendritic spines involving learning and extinction. Our results reveal a finely coordinated mechanism that jointly leverages the spatiotemporal organization and plastic patterns of dendritic spines to support dendritic signal integration and tone discrimination in a learning-dependent manner.

## MATERIALS AND METHODS

### Experimental design

#### Animals

All experiments were approved and conducted in accordance with the University of Hong Kong Committee on the Use of Live Animals in Teaching and Research (CULATR 4262-17) guidelines and under license from the Hong Kong SAR Government’s Department of Health. Eight- to 10-week-old C57BL/6J mice (the Jackson Laboratory) were used for awake calcium imaging. Mice were group housed in a 12-hour light on/12-hour light off schedule in The Laboratory Animal Unit, The University of Hong Kong, accredited by Association for Assessment and Accreditation of Laboratory Animal Care International. Food and water were provided ad libitum.

#### Virus injection

A mixture of AAV9.CaMKII-Cre, AAV9.Flex.Syn.GCaMP7s, and AAV9.Flex.tdTomato (2.1 × 10^13^, 2.5 × 10^13^, 2.1 × 10^13^ genome copies/ml, respectively, Addgene) was used at ∼1:22,000:10,000 to drive the expression of GCaMP7s and tdTomato in L5 PNs in the right FrA. Specifically, 100 nl of AAVs was unilaterally delivered to the right FrA [AP (anteroposterior): +2.80 mm; ML (mediolateral): −1.00 mm; DV (dorsoventral): −0.75 mm] at 100 nl/min through Neuros Syringes (Hamilton; 33G, blunt end) driven by a KDS Legato 130 syringe pump (KD Scientific). To avoid backflow, the needle stayed in place for 10 min after injection. P30 mice were used for intracranial AAV injection. The cranial window was implanted around 18 days after injection. The first imaging session was around 28 days after injection.

#### Cranial window

Cranial window surgery was performed on C57BL/6J mice after 2 weeks of AAV injection. Mice were anaesthetized with ketamine/xylazine intraperitoneally at 6 μl/g body weight. Drugs were administered before the surgery including carprofen (subcutaneous injection; 5 mg/kg, Zoetis UK Ltd.) to reduce inflammation and dexamethasone (intramuscular injection; 2 mg/kg, Sigma-Aldrich) to minimize brain swelling. The scalp was removed, and a metal head plate (Neurotar) was attached to the skull by cyanoacrylate glue and secured with dental cement (C&B Metabond) and an anchor screw (M1 × 3-mm length, contralateral to the imaging side on interparietal bone without penetrating into the brain). A 2 mm–by–2 mm square craniotomy was made on the right FrA. The dura over the FrA was removed by fine forceps (Fine Science Tools). A #2 cover glass (Electron Microscopy Sciences) of the same size was glued to the edges of the bones from the craniotomy by tissue adhesive (Vetbond, 3M Company). The cortex was immerged to artificial cerebrospinal fluid during the entire process before adding the tissue adhesive. Last, the glass was secured by adding another layer of cyanoacrylate gel and dental cement. Mice were monitored under a heat lamp during recovery from the anesthesia, and subcutaneous injection of buprenorphine (0.1 mg/kg; Indivior UK Ltd.) was administered to reduce pain. For postoperative care, mice were injected with carprofen (intraperitoneal injection, 5 mg/kg) and buprenorphine (subcutaneous injection, 0.1 mg/kg) twice daily for 5 days. Baytril enrofloxacin (Bayer, 0.05% oral solution) and Rimadyl (Bio-Serv, oral tablet) were provided to prevent microbial infection and inflammation, respectively. A minimum 10-day recovery period after surgery was required before imaging.

#### In vivo two-photon imaging

Freely behaving mice were head fixed on an air-lifted platform (Mobile Home Cage, Neurotar). Before imaging, mice were habituated to the setup. The mouse’s head was fixed to the clamp on the arm of the Mobile Home Cage through a metal head mount (Neurotar) in a carbon fiber cage (diameter, 180 mm). The mouse in the cage was lifted by an air compressor (Leister Technologies AG) in the next room to minimize noise. The images were acquired using a two-photon microscope (Olympus FVMPE-RS) with a water immersion lens (25×; numerical aperture, 1.05; Olympus) and two Coherent Chameleon Vision II lasers. The GCaMP channel was acquired with a 920-nm laser and emission dichroic mirror DM560; the tdTomato channel was acquired with a 1080-nm laser, emission dichroic mirror DM560, and long-pass filter LP590. Synchronization of imaging and tone was achieved by an analog program (Olympus) connected to a sound generator (Coulbourn Instruments, A12-33) with a speaker module (Coulbourn Instruments). The frequency and amplitude were adjustable on the sound generator. To set the duration and delay time for the tone, a waveform generator (Siglent, sdg-2042x) was used. The baseline tone response was acquired 2 days before FC. Both calcium sensor and structural fluorescent signals were captured on a single focal plane at a frame rate of 1 Hz under resonant mode. Shaft signals were recorded simultaneously with spine activity on the same plane. ROI variability was to maximize dendritic coverage versus image resolution. The time series contained a 5-s pretone and 10-s tone (4 or 12 kHz) presentation period per ROI. After tone playing, high-resolution images of dendritic spines were acquired under Galvo mode as a *z*-stack. The intertrial period of each tone play was 1 min, and the total imaging time was less than 30 min.

With the sparse labeling of the dendrites, the same ROIs were identified on the basis of brain vasculature and the morphology of the dendritic branch from 3D imaging stacks obtained in the first imaging session, as described previously (*12*). Dendritic branches from L5 PNs were imaged at a 0- to 100-μm distance below the pia surface. The same dendritic segments were identified from 3D stacks taken at different time points with high image quality (ratio of signal to background noise >4:1). The images of the target dendritic segments were compared across imaging sessions on the basis of existing spines and the curvature of the dendritic shafts. The number and location of dendritic protrusions (protrusion lengths were more than one-third of the dendritic shaft diameter) were identified. Filopodia were identified as long, thin structures (generally larger than twice the average spine length, ratio of head diameter to neck diameter <1.2:1, and ratio of length to neck diameter >3:1) (*12*). The remaining protrusions were classified as spines. Filopodia were excluded, and no subtypes of spines were distinguished in our analysis. 3D stacks were used to ensure that tissue movements and rotation between imaging intervals did not hinder spine identification. Spines were considered as identical between views if their positions were unchanged with respect to adjacent landmarks. Spines were considered different if they were more than 0.7 μm away from the first view. Spine formation was identified as new spines formed that did not exist from the immediately preceding session. Meanwhile, spine elimination refers to the spine that exists on one imaging session and disappear on the following session.

#### Auditory-cued FC and fear extinction

After recovery from surgeries, all mice were habituated to the Mobile Home Cage setup. The mouse’s head was fixed to the clamp on the arm of the Mobile Home Cage through a metal head mount (Neurotar) in a carbon fiber cage (diameter, 180 mm). The mouse in the cage was lifted by an air compressor (Leister Technologies AG) in the other room to minimize noise. After at least 3 days of habituation, mice were subjected to paired FC or unpaired training 1 day after the first imaging time point using the FreezeFrame system with a sound-attenuating chamber (Coulbourn Instruments). The tone and footshock were controlled by the preset program in Actimetrics FreezeFrame software (version 4.01; Coulbourn Instruments). The FC chamber was equipped with stainless-steel shocking grids connected to a precision feedback current–regulated shocker (Coulbourn Instruments) during training and with plastic nonshocking test grids for fear testing. Behavior was recorded using low-light video cameras.

The cage was transferred to the room at least 1 hour for habituation before FC training and tests. Mice were allowed to explore the content for 1 min on the shocking grid (context A: shocking floor and ethanol scent), followed by 30-s, 4000-Hz, 80-dB auditory cue (CS) coterminated with 2-s, 0.5-mA scrambled footshock (US). For the unpaired group, the tone and shock were randomly distributed. The intertrial interval was 20 s among the three trials, and the mice were returned to their home cages after 1 min. For extinction training, mice were presented with five 120-s CS presentations with 120-s intertrial intervals each day for two consecutive days. The freezing rate in the last extinction trial at D5 was measured as the freezing response at D5. Only the extinction group will be subject to extinction training. The percentage of freezing was calculated by FreezeFrame software with confirmation of the threshold manually through the recorded videos.

#### Experimental timeline

As illustrated in Fig. 1E, after the surgery recovery (∼10 days) and habituation to the Mobile Home Cage setup, mice were subjected to the baseline image session (D0), where the dendrites of L5 PNs with clear morphological display were randomly selected. We recorded the responses of dendrites to both CS and control 12-kHz tone. On the next day, mice were subjected to either paired FC or unpaired training in the FC chamber. Within 1-hour post-FC, the D1 image session was conducted for the dendrites identified at D0 for recording their response to both tones. Two days later at D3, the same dendrites were imaged, and mice were then subjected to fear recall test in the recall chamber. At D4 and D5, mice in the extinction group further received extinction training, and the rest mice stayed in the home cage. Mice were imaged within 1 hour after D5 extinction or recall.

### Calcium imaging analysis

#### Processing of single spine’s calcium data

The time-lapse *z*-stack of single-plane GCaMP7s and structural labeling was registered using the FIJI plug-in StackReg (*76*) for motion correction. On the basis of the imaging of the map for soma tracking purpose and the density of the dendritic spines, the stacks were classified to L2/3 or L5 categories where L2/3 apical dendritic spines have a higher density than those from L5 PNs (*77*). The ROIs of each spine were manually assigned using the Matlab open source FluoroSNNAP (*78*). Structural labeling was used as a reference for the manual selection of ROIs. All spines were included for the given dendritic segment regardless of the brightness of the GCaMP7 signal. The criteria of identifying dendritic spines were applied as in a previously published paper (*12*). Calcium transients were calculated using the formula *dF*/*F* = (*F* − *F*_0_)/*F*_0_ (with *F*_0_ is the 20th percentile of mean fluorescence during the entire trace) and used for all analyses of Ca^2+^ activity. Calcium imaging analysis was performed by Matlab.

#### Analysis of synchronous and nonsynchronous spine pairs

To identify synchronous spine pairs, the calcium traces of each pair of spines in the same dendrite were analyzed with two-tailed Pearson’s correlation test with FDR correction. If data of the spine pair were positively and significantly correlated (*P* < 0.05), this pair of spines was identified as temporarily synchronous spines; otherwise, the pair of spines was nonsynchronous spines. The spine synchronous probability was calculated by

$$S_{\text{spine}}=\frac{N_{\text{syn}}}{N_{\text{den}}-1}\times 100\%$$

where $S_{\text{spine}}$ is the spine synchronous probability of the target spine, $N_{\text{syn}}$ is the number of synchronous spines of the target spine, and $N_{\text{den}}$ is the number of spines in the dendrite. In addition, dendrite coherency was calculated by

$$C_{\text{den}}=\frac{N_{\text{syn}-\text{pair}}}{N_{\text{pair}}}\times 100\%$$

where $C_{\text{den}}$ is the dendrite coherency, $N_{\text{syn}-\text{pair}}$ is the number of synchronous spine pairs in the dendrite, and $N_{\text{pair}}$ is the total number of possible spine pairs in the dendrite.

To exclude the potential possibility that the spines’ synchronicity on a dendrite was induced by the excitation of the dendrite per se, we also analyzed the spines whose synchronous probability was lower than 100% only (fig. S3, A to C).

The intrapair distance was calculated inside the synchronous spine pairs or nonsynchronous spine pairs. The simulation of distance between synchronous spines was performed on Matlab and based on the corresponding real location information of dendritic spines. Upon each spine, we randomized the synchronous possibility with the rest spines on the same dendrite to simulate the random synchronous spine pairs. The simulation was conducted for 1000 times, and the intrapair distance was calculated for the simulated synchronous spine pairs.

To verify the clustering feature of different types of dendritic spines (Fig. 5, F to H), a custom-written Matlab program was used. The simulation was performed on the basis of the real dendritic shaft length and the number of targeted spines. The cumulative distribution function analysis of the closest distance between the targeted spines was subsequently performed on the simulated data (1000 times of repeats) and real data.

### Simulation of the biophysically detailed neural network model

#### Basic setting of the simulation

The computational neural network model was modified on the basis of the previous publications (*39*, *40*). The model was developed on the parallel NEURON simulation platform (*79*) and NetPyNE (Networks using Python and NEURON) (*80*) package tool. The network includes conductance-based simplified cell models with parameters optimized to reproduce physiological phenomena like dendritic spine plasticity. The detailed simulation parameters and configuration settings are available in the project’s GitHub repository. In the cortical region of the network, we modeled 20 excitatory PNs, 10 inhibitory PVs, and 10 SOMs. In addition, we used the spike generator “VecStim” objects in NetPyNE to simulate the stimulation (20 “CS” neurons, 20 “12-kHz tone” neurons, and 20 “shock” neurons) from long-projecting neurons to cortical PNs. The local connectivity (presynaptic to postsynaptic) was made between PN and PV, PN and SOM, PV and PV, PV and PN, PV and SOM, SOM and SOM, SOM and PN, and SOM and PV.

On the simulated dendrite, we set the candidate spine position every 1 μm along the dendrite. The range of dendritic spine weights is 0 to 15, and the physical spine threshold is set to 3: When the weight is less than 3, the spine is considered physically absent; when it is greater than 3, the spine is considered physically present. Hereafter, the “spine” in the neural network will denote a physically present spine, unless explicitly stated otherwise. We randomly generated the weights for all candidate spine positions so that the overall spine density is ∼40 per 100 μm. After the initial settings of synaptic connections and the allocation of randomly generated weights into synapses, the simulation of network went through the associative learning session, sleep session (consolidation), and recall test session. During the associative learning session, the long-projecting stimuli of CS and shock were administered together, with the activation of STDP for the cortical neurons (*80*, *81*). STDP was inactive during the other two sessions. In the sleep session, homeostatic synaptic plasticity (HSP) was introduced with a constant 5-Hz stimulation on synapses to mimic the homeostatic synaptic scaling during memory consolidation (*82*). During the recall test session, HSP and STDP were both inactive, and only the activities and weights of dendrites and spines were recorded.

#### Simulation of spine synchronicity

For the simulation of spine synchronicity, we first identified reference spines by randomly selecting 5 spines from each of the 20 PNs. For each of 100 reference spines, we defined a local dendritic neighborhood spanning ±50 μm along the shaft. Within this range, we used Pearson’s correlation test to assess activity relationships. Spines exhibiting a significant positive correlation with the reference spine were classified as “synchronous,” while all others were labeled as “nonsynchronous.” To control for differences in group size, we matched the number of stimulated synchronous spines to the number of stimulated nonsynchronous spines by selecting the lower of the two group counts. Thus, the same number of synchronous or nonsynchronous spines that were positioned closest to the reference spine will be stimulated. This classification method mirrors the criteria applied to our in vivo experimental data. We then simulated the simultaneous activation of either the synchronous or nonsynchronous spine populations along with their corresponding reference spine. To isolate the impact of these specific inputs, stimulations were performed in a temporally separated manner, preventing interference from other stimulations on the same neuron. Both local (±50 μm from the reference spine) and branch (whole dendritic branch) activities were recorded to assess the computational output.

#### Simulation of spine elimination

For the simulation experiment on spine elimination, we first defined “eliminated spines” as those present before the associative learning session (weight >3) but absent (weight <3) during the recall test, the standard that is consistent with the experimental protocols. To artificially rescue spine elimination, we first quantified the weight loss of the eliminated spines by calculating the difference between its prelearning baseline weight and its weight at the recall session by calculating the difference between its prelearning baseline weight and its weight at the recall session. This calculated weight was then computationally reinstated to the eliminated spine, restoring its synaptic strength to the prelearning state. As for random restoration control, the reduction of synaptic weight of eliminated spines from the baseline to recall was added to a randomly selected population of surviving spines. This control simulation was repeated 120 times to ensure statistical robustness. Following both the targeted restoration and control manipulation, a simulated recall test was performed, and the resulting dendritic activity was recorded.

### PCA of dendritic activities

To compare the dendritic pattern toward CS versus 12-kHz tone, the calcium activity traces of dendritic shafts from all imaging sessions were collected. PCA was performed on all aligned dendritic calcium data, and the top three components (PC1, PC2, and PC3) with the highest PCA scores were selected to describe the pattern of the dendritic responses to stimuli (Fig. 6, J to L). Then, the Euclidean distance in the PC1-PC2-PC3 3D space between pairwise dendritic response patterns to CS and 12-kHz tone was measured as the discrimination index at each imaging session. The discrimination changes between two behavioral sessions were then calculated by the differences in discrimination indexes in two sessions. All analyses and plots were made in Matlab.

### Statistical analysis

GraphPad Prism software 9.0 (GraphPad Software) and Matlab with a significance level set at 0.05 were used to perform statistical analysis. Data are presented as the means ± SD or means ± SEM, unless otherwise stated. All box-whisker plots are with box limits of the first and third quartiles, a line inside the box of the median, and whiskers of data range. For most comparisons including nested data formats (e.g., spines from dendrites from mice), the linear mixed-effects model (using Matlab “fitlme”) was used. For the correlation analysis, the linear mixed-effects model was used to quantify the association between variables. Statistical significance is reported as the *P* value for the fixed-effect term of interest, along with its estimated regression coefficient (β). This model considers the data dependence for the repeated measurements from same dendrite or same animal when computing the statistical significance, and it is more stringent than simply computing it over all data (*83*). Here, we fitted random intercepts for each comparison, and the random effects and reported *P* values for the model were listed (table S1). For paired comparisons in Fig. 3, a two-sided paired *t* test was used in Matlab (“ttest” function). To compare the distance between spine populations, a two-sample Kolmogorov-Smirnov test (“kstest2” function) with FDR correction was made between simulated data and real data on Matlab. For the analysis of the relationship between spine elimination fate and spine activities (fig. S4), the logistic model was performed in Matlab. For the causal inference analysis (Fig. 4), the two-stage least squares model was used as previously described (*48*, *51*). The first-stage regression was given by

$$\left\{ \begin{array}{c} X_{1}=a_{10}+a_{11}Z_{1}+a_{12}Z_{2}+\epsilon_{1}\\ X_{2}=a_{20}+a_{21}Z_{1}+a_{22}Z_{2}+\epsilon_{2} \end{array}\right.$$

where $Z_{1}$ is the CS response of the target spine, and $Z_{2}$ is the sum of CS responses of its neighboring spines at the D0 baseline session; $X_{1}$ is the CS response of the target spine, and $X_{2}$ is the sum of CS responses of its neighboring spines in the D1 imaging session; $a_{10}$ and $a_{20}$ are intercepts; $a_{11}$, $a_{12}$, $a_{21}$, and $a_{22}$ are coefficients; and $\epsilon_{1}$ and $\epsilon_{2}$ are error terms. The regressed results of $X_{1}$ and $X_{2}$ were then used in the second-stage regression given by

$$Y=\frac{\text{exp}\left(b_{0}+b_{1}X_{1}+b_{2}X_{2}+\epsilon \right)}{1+\text{exp}\left(b_{0}+b_{1}X_{1}+b_{2}X_{2}+\epsilon \right)}$$

where Y is the elimination fate of the target spine, $b_{0}$ is the intercept, $b_{1}$ and $b_{2}$ are coefficients, and ϵ is the error term. The coefficients and *P* value in each stage and the overall *P* value are calculated and listed in table S1.

For the unpaired comparisons in fig. S8, the Shapiro-Wilk test was used to test for the normality of all datasets. An *F* test was used to test for the homogeneity of variance of two groups, while the Brown-Forsythe test was used to test for the homogeneity of variance of three or more groups. The two-tailed Mann-Whitney test and Kruskal-Wallis test with post hoc Dunn’s multiple comparisons test were used. Unless otherwise stated, all statistical tests were two-tailed, and *P* values less than 0.05 were considered as statistically significant.

## Supplementary Materials

### This PDF file includes:

Supplementary text Figs. S1 to S10 Tables S1 and S2 References
