# Supplementary material for: Linking functional and structural dendritic spine remodeling during fear learning and extinction in vivo
Source: Sci Adv. 2026 Jul 17;12(29):eaec3961. doi: 10.1126/sciadv.aec3961 (PMC13378561; doi:10.1126/sciadv.aec3961)
Supplement: Supplementary file 1 — Supplementary text Figs. S1 to S10 Tables S1 and S2 References [file sciadv.aec3961_sm.pdf]

Science Advances
AAAS

Supplementary Materials for

Linking functional and structural dendritic spine remodeling during fear learning and extinction in vivo

Xiaoyang Li *et al.*

Corresponding author: Cora Sau Wan Lai, coraswl@hku.hk

Sci. Adv. **12**, eaec3961 (2026)
DOI: 10.1126/sciadv.aec3961

This PDF file includes:

Supplementary text
Figs. S1 to S10
Tables S1 and S2
References

Supplementary information on methodology

To minimize photobleaching across the longitudinal design of this study, we used 1 Hz acquisition for the experiment, which includes four imaging timepoints and two tone-evoked recordings per timepoint (eight sessions in total). Under these repeated-imaging conditions, pilot datasets acquired at higher frame rates showed substantial signal loss across sessions, whereas the 1 Hz protocol maintained stable fluorescence levels across the multi-day imaging period. To further reduce motion-related artifacts, we simultaneously acquired a functional channel (GCaMP) and a structural channel (tdTomato) and used resonant scanning with averaging across two frames per channel (effective ~150 ms per scan frame). This imaging strategy was selected to balance longitudinal signal stability with sufficient sensitivity to capture spine calcium activity.

To directly address the concern that 1 Hz may undersample GCaMP7s dynamics, we performed a validation experiment using the same indicator but acquired at 15 Hz (resonant scanning with comparable frame averaging). We then (i) applied the same spine synchronicity analysis pipeline to the 15 Hz data and the downsampled 1 Hz data, (ii) analysed the binarized co-events (co-activity) based on the 15 Hz data, and (iii) compared correlation p-value matrixes and co-activity ratios of synchronous spine pairs across the two sampling rates. Fig. S1A showed an example dendrite with 15 Hz imaging as described and we performed additional downsampling procedure to get 1 Hz data (fig. S1B). We then performed pairwise correlation analyses among spines and got the p-value matrix (fig. S1C), allowing identification of "synchronous" (i.e., significantly correlated) spine pairs (or spine synchronicity). We found that the correlation analyses in 1 Hz data do not inflate estimates of spine synchronicity relative to 15 Hz; instead, the 1 Hz analysis yielded a more stringent classification while still capturing shared activity trends over time.

We then detected calcium events using the algorithm described in the prior study *(27)*: Briefly, noise was estimated for each smoothed dF/F trace and the 2 times of noise value was used for detecting active portions. Active portions were extended to the baseline crossing (0.5 times the noise value) and refined by finding local peaks to separate temporally overlapping events ("piggybacking"). We detected calcium events from the smoothed 15 Hz traces and binarized these events to quantify event overlap ("co-activity") between spine pairs (red marks in fig. S1B). We showed that, using either Pearson's correlation or Pearson's correlation with FDR correction to define synchronous pairs, significantly correlated spine pairs (synchronous pairs) exhibited higher co-activity ratios than non-synchronous pairs (fig. S1C-D), supporting the validity of the correlation-based coupling metric. Accordingly, we used the term, "spine synchronicity" to reflect that our primary measure captures correlation of activity fluctuations over time rather than strict event-level synchrony, and we adopted Pearson's correlation with FDR correction to reduce false positives in multiple pairwise testing in the Figure. 2 in the manuscript.

The imaging sessions were performed in different context from the behavioral setup in the current study. To confirm that the neural responses measured during imaging are behaviorally relevant, we evaluated the pupil dilation response of mice in the head-fixed imaging context (Mobile Home Cage setup) after behavioral experiments (fig. S2G-H) *(84)*. Our data show that mice exhibited significant pupil dilation during CS probe sessions on Day 1 (fear conditioning) and Day 3 (recall) in the head-fixed context, whereas a non-CS control tone (12 kHz) did not evoke significant pupil changes (Fig. S2G). After extinction (Day 5), CS presentation no longer produced a significant pupil dilation response (fig. S2G), consistent with reduced fear expression. Importantly, in this same cohort, freezing levels measured in the behavioral chamber during the recall test were significantly correlated with the magnitude of CS-evoked pupil dilation measured in the head-fixed context (fig. S2H). Together, these data support that the tone-evoked responses measured during head-fixed

probe sessions reflect a conditioned fear response, thereby strengthening the interpretation that CS-evoked activity patterns quantified during imaging are behaviorally relevant.

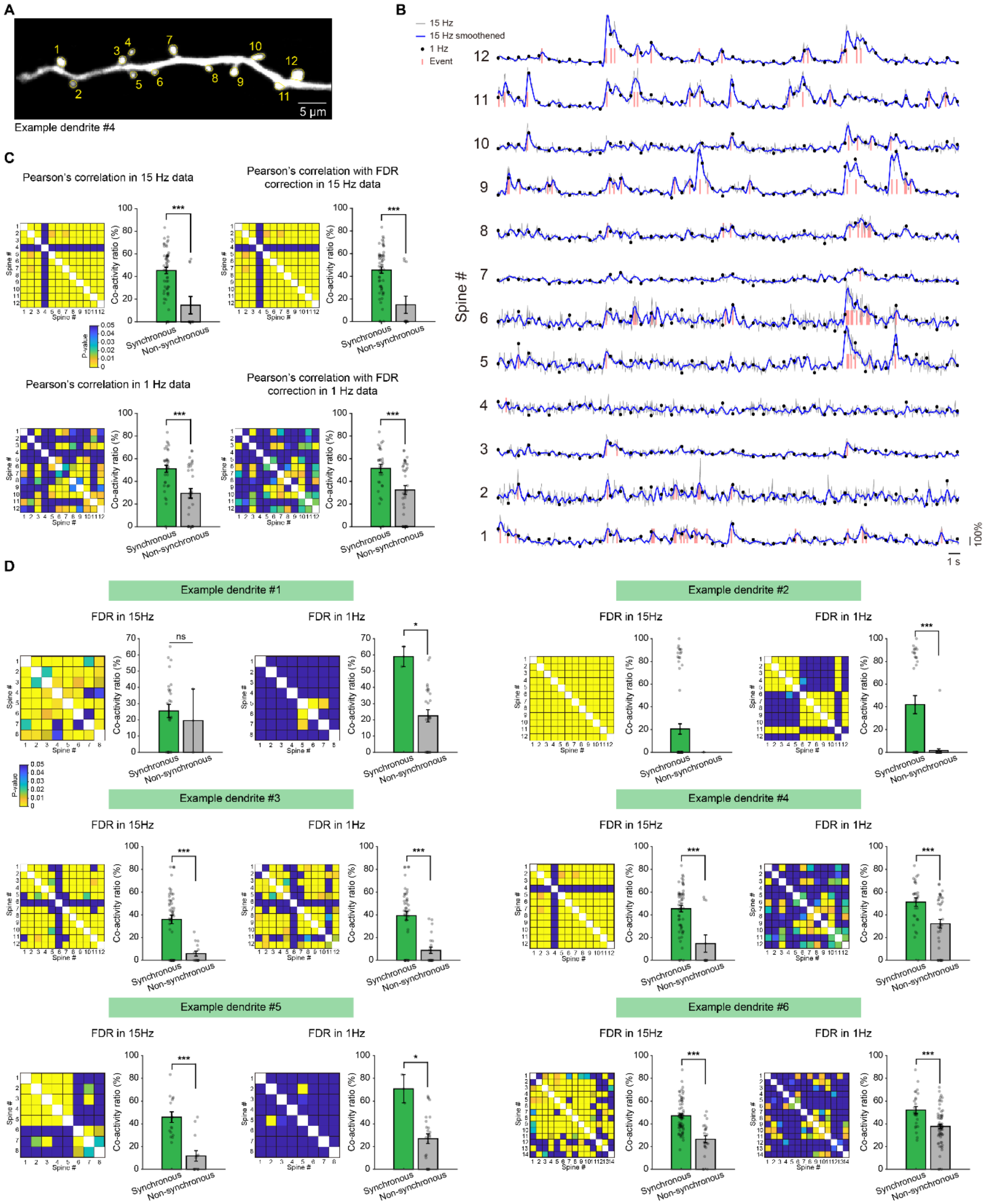

Fig. S1. ***Comparisons of imaging framerates and analyses methods.*** (**A**) The average projection of tdTomato (structural channel) from a representative dendritic segment acquired under 15 Hz resonant scanning. Scale bar, 5 µm. (**B**) Traces of dF/F from the corresponding spines labeled in (A). Gray, raw 15 Hz $\Delta F/F$; blue, 15 Hz traces smoothed with a Savitzky–Golay filter (1 s window, polynomial order 4); black dots, the same traces downsampled to 1 Hz; red shaded regions, calcium events detected from the smoothed 15 Hz traces. Events were detected using the algorithm described in Wright et al. (27): Briefly, noise was estimated for each smoothed dF/F trace and the 2 times of noise value was used for detecting active portions. Active portions were extended to the baseline crossing (0.5 times the noise value) and refined by finding local peaks to separate temporally overlapping events ("piggybacking"). Scale bars, 1 s (horizontal) and 100% dF/F (vertical). (**C**) Co-activity ratio for synchronous versus non- synchronous spine pairs defined by

Pearson's correlation (left) or Pearson's correlation with FDR correction (right), computed separately for 15 Hz data (top) and 1 Hz data (bottom). Heatmaps show the pairwise correlation significance (p-value) matrix used to classify synchronous spine pairs. Co-activity was quantified as temporal overlap of binarized calcium events between two spines; continuous overlapping frames were counted as a single co-activity to avoid double counting. Co-activity ratio was calculated as co-event count divided by the total event count for the pair. Sample sizes (spine pairs): $n = 55$ (15 Hz Pearson, synchronous), $n = 55$ (15 Hz Pearson+FDR, synchronous), $n = 33$ (1 Hz Pearson, synchronous), $n = 23$ (1 Hz Pearson+FDR, synchronous); total analyzed pairs, 66. (**D**) Same analysis as in (C) using Pearson correlation with FDR correction, shown for six additional dendrites (example dendrites #1-#6), comparing co-activity ratios between synchronous and non-synchronous spine pairs at 15 Hz and after downsampling to 1 Hz. FDR correction follows the standard Benjamini–Hochberg procedure.

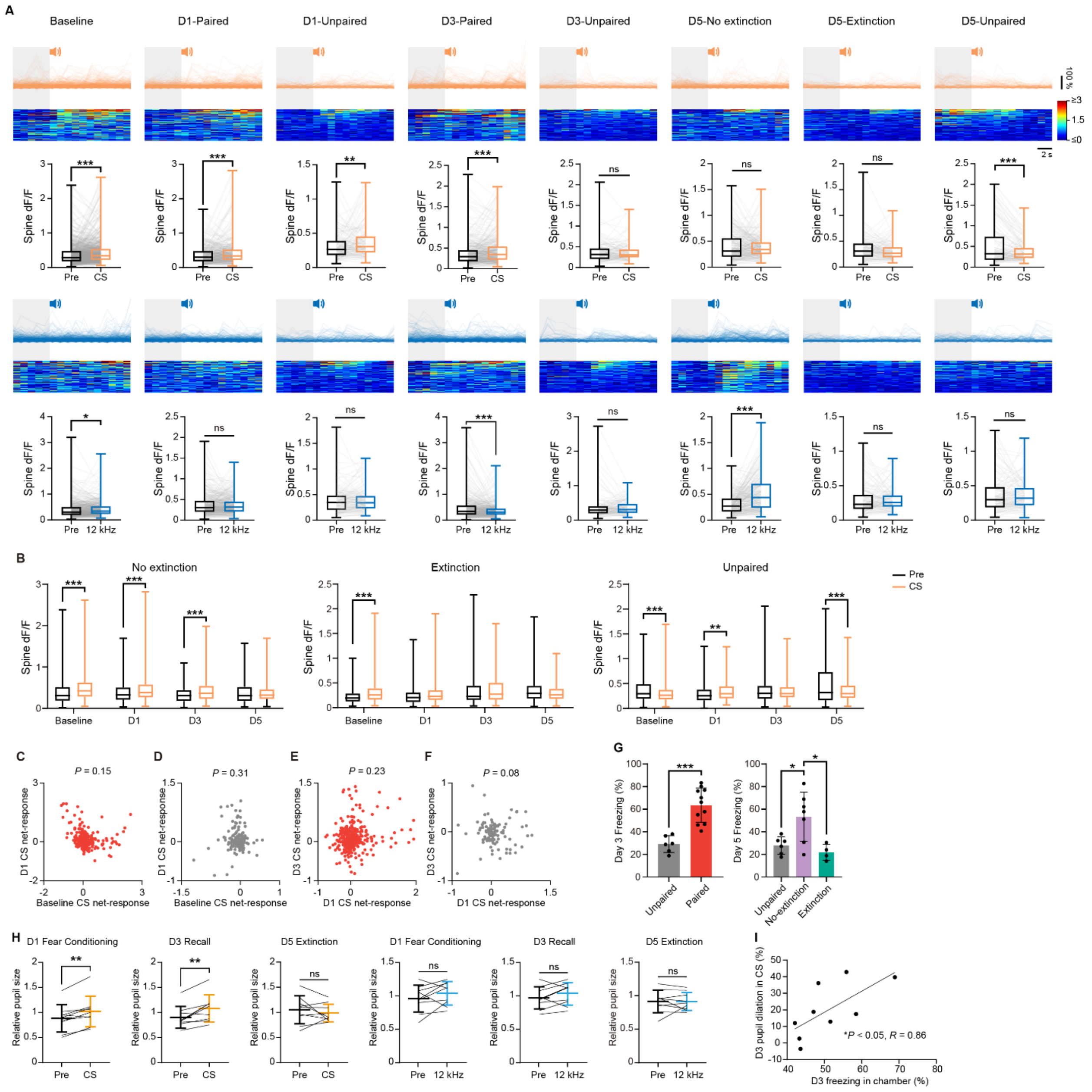

Fig. S2. ***Overall spine population and behavioral response to stimuli.*** (**A**) Top: spine calcium signals in response to CS or 12 kHz tone. Middle: heatmaps show the calcium dynamics of dendritic spines. Bottom: Quantification of the mean calcium dF/F during pre-tone period (Pre) versus tone-playing period (CS or 12 kHz) of all imaged dendritic spines. Data shown as box-whisker plots (box limits: the first and third quartile; line inside box: median; whiskers: data range); each grey line represents the calcium signal change in one dendritic spine; *n* = 623 spines from 49 dendrites from 14 mice (baseline); *n* = 454 spines from 37 dendrites from 11 mice (D1-Paired); *n* = 157 spines from 12 dendrites from 3 mice (D1-Unpaired); *n* = 430 spines from 37 dendrites from 11 mice (D3-Paired); *n* = 144 spines from 12 dendrites from 3 mice (D3-Unpaired); *n* = 275 spines from 23 dendrites from 7 mice (D5-No extinction); *n* = 143 spines from 14 dendrites from 4 mice (D5-Extinction); *n* = 137 spines from 12 dendrites from 3 mice (D5-Unpaired). (**B**) spine calcium signals to CS in each subgroup. Linear mixed-effects model. (**C** to **F**) Linear mixed-effects model testing the correlation of CS net-response between two different sessions with estimated regression coefficient (β); *n* = 376 spines from 37 dendrites from 11 mice (B); *n* = 128 spines from 12 dendrites from 3 mice (C); *n* = 349 spines from 37 dendrites from 11 mice (D); *n* = 114 spines from 12 dendrites from 3 mice (E). (**G**) Quantification of freezing rate

during the recall test on Day 3 (left) and on Day 5 (right). The freezing rate in the last extinction trial on Day 5 was measured as the freezing response in Day 5. Data shown as mean ± SD. Left: ***P < 0.001; unpaired t-test; n = 6 mice for unpaired group; n = 11 mice for paired group. Right: *P < 0.05; one-way ANOVA followed by Tukey's multiple comparison test; n = 6 mice for unpaired group; n = 7 mice for no-extinction group; n = 4 mice for extinction group. In the unpaired group, three animals did not produce imaging data of sufficient quality and were therefore excluded from the imaging analyses. (**H**)Relative pupil size measured during head fixation on the Mobile Home Cage setup, quantified in the 10 s baseline period before tone onset ("Pre") and during the 10 s tone presentation ("CS" or control tone). Pupil size was normalized to the mean pupil size during the 1-min period prior to any tone exposure. Sample size in mouse: n = 9. **P < 0.01, paired t-test. Data shown as mean ± SD. (**I**) Two-tailed Pearson's correlation analysis between CS-evoked pupil dilation in Mobile Home Cage context and the freezing rate in measured during the Day 3 recall test in the behavioral chamber. The pupil size dilation ratio was calculated as: (size in CS – size in Pre) / size in Pre.

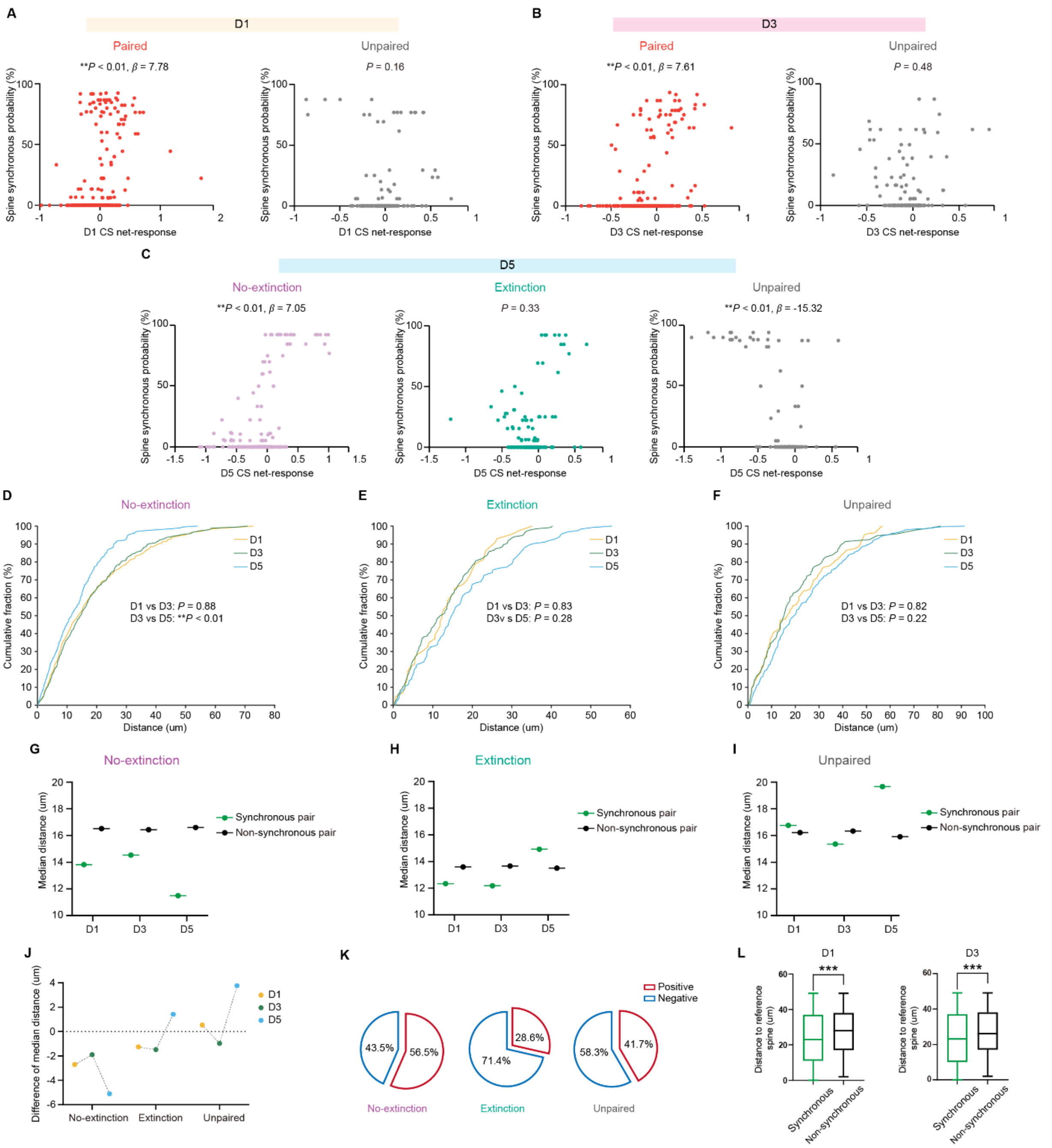

Fig. S3. ***Properties of synchronous spine pairs.*** (**A** to **C**) Linear mixed-effects model testing the correlation between the synchronous probability and CS net-response of spine (excluding spines with 100% synchronous probability) in different periods; *n* = 418 spines from 37 dendrites from 11 mice (A, paired), *n* = 148 spines from 12 dendrites from 3 mice (A, unpaired), *n* = 374 spines from 37 dendrites from 11 mice (B, paired), *n* = 144 spines from 12 dendrites from 3 mice (B, unpaired), *n* = 270 spines from 23 dendrites from 7 mice (C, no-extinction), *n* = 143 spines from 14 dendrites from 4 mice (C, extinction), *n* = 116 from 12 dendrites from 3 mice (C, unpaired). (**D** to **F**) Cumulative distribution function of the intra-pair distance between synchronous spines at different sessions. The two-sample Kolmogorov-Smirnov test with FDR correction was performed between D1 versus D3 and D3 versus D5; *n* = 408 pairs from 23 dendrites from 7 mice (D, D1), *n* = 335 pairs from 23 dendrites from 7 mice (D, D3), *n* = 204 pairs from 23 dendrites from 7 mice (D, D5), *n* = 43 pairs from 14 dendrites from 4 mice (E, D1), *n* = 134 pairs from 14 dendrites from 4 mice (E, D3), *n* = 123 pairs from 14 dendrites from 4 mice (E, D5), *n* = 135 pairs from 12 dendrites from 3 mice (F, D1), *n* = 93 pairs from 12 dendrites from 3 mice (F, D3),

n = 250 pairs from 12 dendrites from 3 mice (F, D5). (**G** to **I**) Median value of the intra-pair distance between synchronous spines and between non-synchronous spines. (**J**) The difference of intra-pair distance median between synchronous spine pair and non-synchronous spine pair (synchronous – non-synchronous). (**K**) Pie chart showing the fractions of dendrites with positive or negative net-response in each group. (**L**) The distance between reference spine to synchronous spines or to non-synchronous spines from computational simulation network. Data shown as box-whisker plots (box limits: the first and third quartile; line inside box: median; whiskers: data range). ***$P < 0.001$, linear mixed-effects model; sample size in spine pair: n = 16744 (D1, synchronous), n = 2626 (D1, non-synchronous), n = 14934 (D3, synchronous), n = 1019 (D3, non-synchronous).

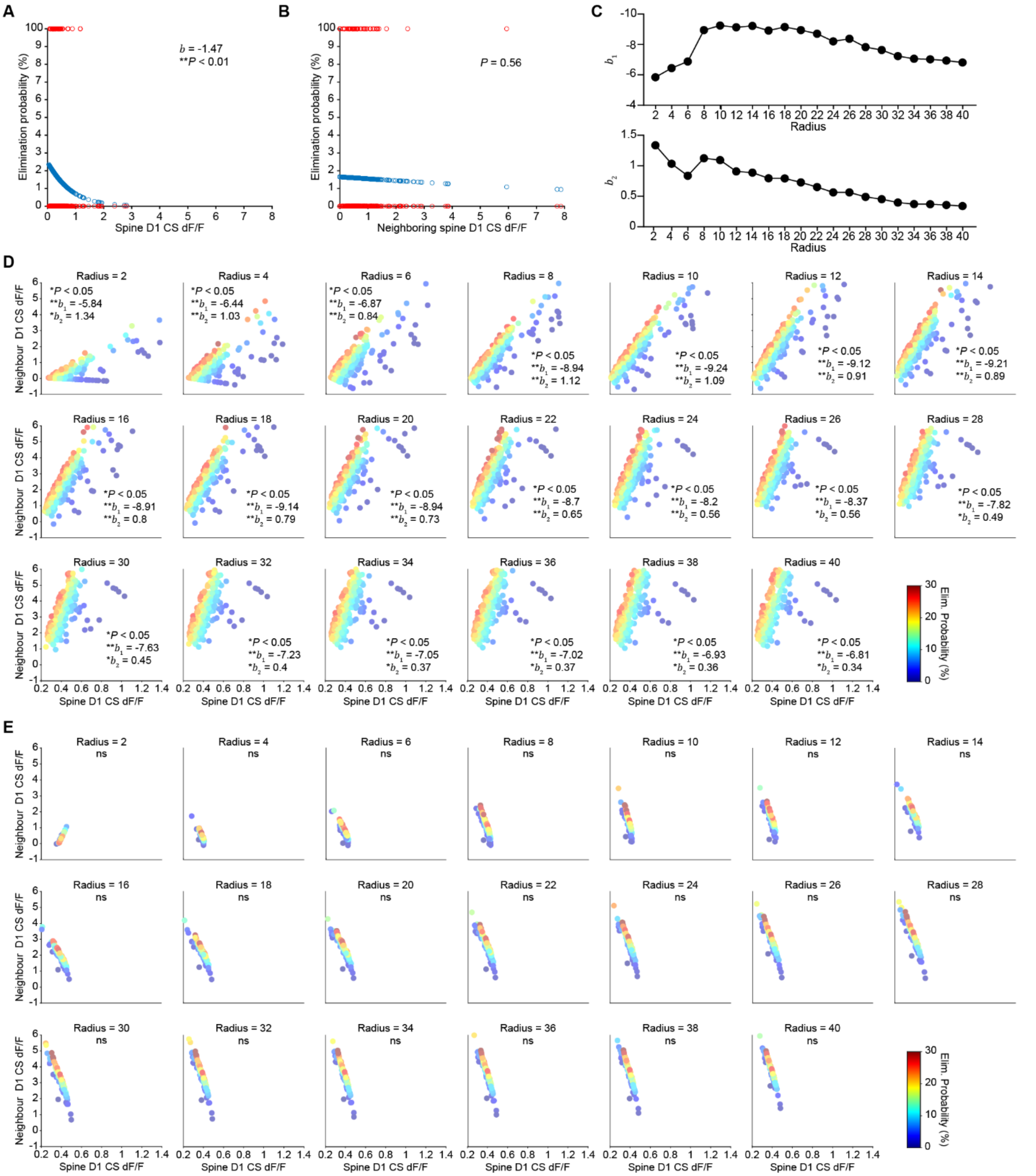

Fig. S4. ***Impact of neighboring spines on elimination remains across measuring radii.*** (**A**) Logistic regression model between the spine elimination fate and spine CS dF/F in D1 in paired group; *b* denotes the coefficient for spine CS dF/F in D1 in the model. (**B**) Logistic regression model between the spine elimination fate and the sum of neighbouring spine CS dF/F in D1 in paired group. The red circles represent the observations of the data, and the blue circles represent the fitted elimination probability response based on observations; n = 128 spines from 37 dendrites from 11 mice (A); n = 128 spines from 37 dendrites from 11 mice (B). (**C**) Coefficients from the two-stage regression model fit across a range of neighbor radii (2–40 µm). Left, estimated coefficient for the spine's own activity term (b_1); right, estimated coefficient for the spine's neighbor-activity term (b_2). Larger radii reduce the magnitude of the neighbor-activity effect, consistent with a spatially local interaction. (**D**) Two-stage regression model fits for

representative radii (2, 4, …, 40 µm). Each point represents a spine. Point color indicates the model-predicted elimination probability. Insets report the significance and fitted coefficients: the spine's own activity term (b_1), the spine's neighbor-activity term (b_2). (**E**) Corresponding plots for the unpaired condition across the same radii, showing no significant (ns) model fit across radii.

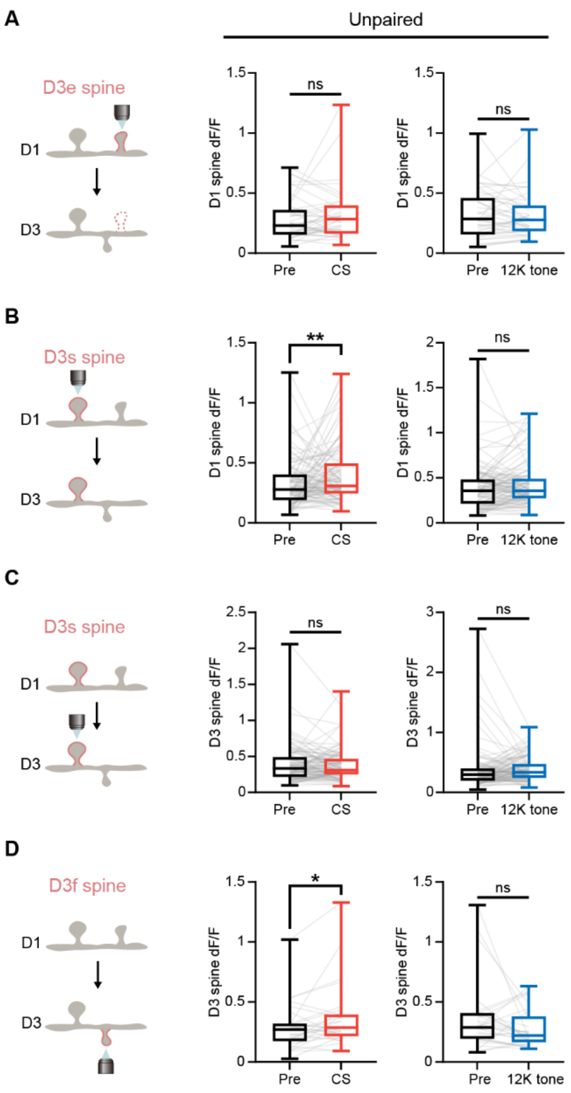

Fig. S5. ***Dendritic spine subgroups in unpaired condition.*** (**A** to **D**) Quantification of the mean calcium dF/F in unpaired group during pre-tone period (Pre) versus tone-playing period (CS or 12 kHz) of D3e spines in D1 FC (A), D3s spines in D1 (B), D3s spines in D3 (C), D3f spines in D3 (D). $*P < 0.05$, $**P < 0.01$, ns: no significant difference; linear mixed-effects model; $n = 43$ spines from 12 dendrites from 3 mice (A), $n = 144$ spines from 12 dendrites from 3 mice (B), $n = 144$ spines from 12 dendrites from 3 mice (C), $n = 30$ spines from 12 dendrites from 3 mice (D). Data shown as box-whisker plots (box limits: the first and third quartile; line inside box: median; whiskers: data range).

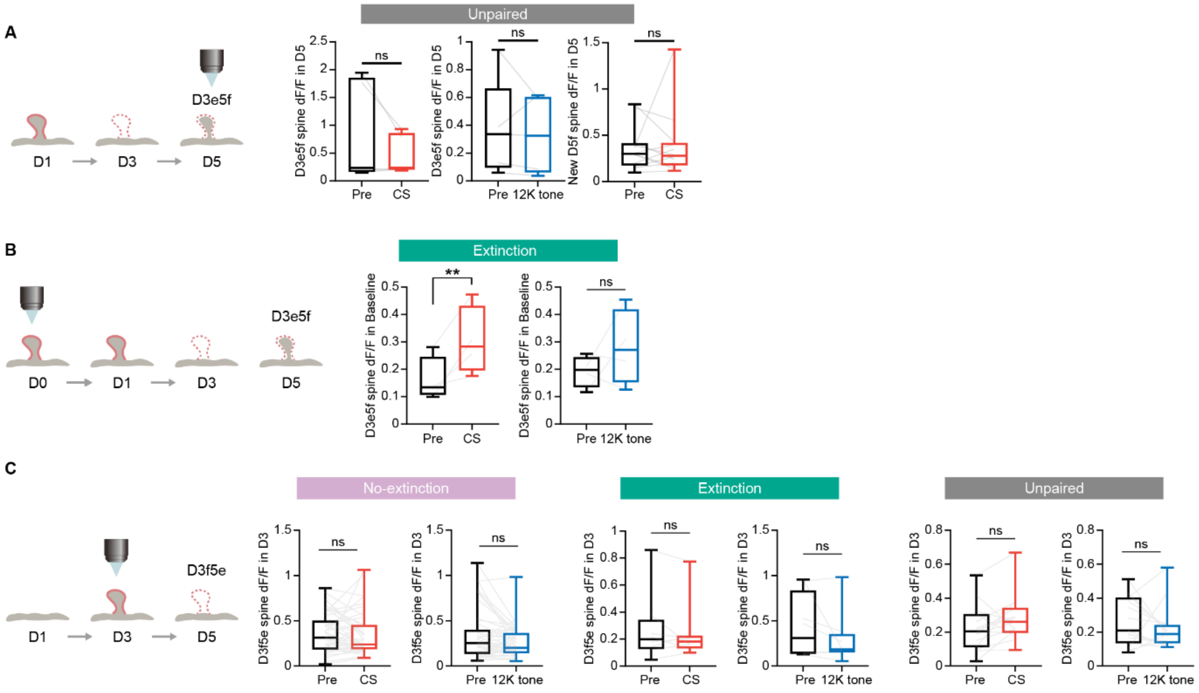

Fig. S6. ***Functional properties of structural remodeling spines.*** *(***A**) Quantification of the mean calcium dF/F during pre-tone period (Pre) versus tone-playing period (CS or 12-kHz tone) of D3e5f spines in D5; ns: no significant difference; linear mixed-effects model; *n* = 5 spines from 12 dendrites from 3 mice (D3e5f); n = 12 spines from 12 dendrites from 3 mice (new D5f). (**B**) Quantification of the mean calcium dF/F during pre-tone period (Pre) versus tone-playing period (CS) of D3e5f spines in baseline. $^{**}P < 0.01$; linear mixed-effects model; *n* = 4 spines from 14 dendrites from 4 mice. (**C**) Quantification of the mean calcium dF/F during pre-tone period (Pre) versus tone-playing period (CS or 12 kHz) of D3f5e spines in D3; ns: no significant difference; linear mixed-effects model; *n* = 36 spines from 23 dendrites from 7 mice (no-extinction), *n* = 8 spines from 14 dendrites from 4 mice (extinction), *n* = 15 spines from 12 dendrites from 3 mice (unpaired). Data shown as box-whisker plots with box limits representing the first and third quartile, the line inside box denoting the median, and whiskers indicating the data range.

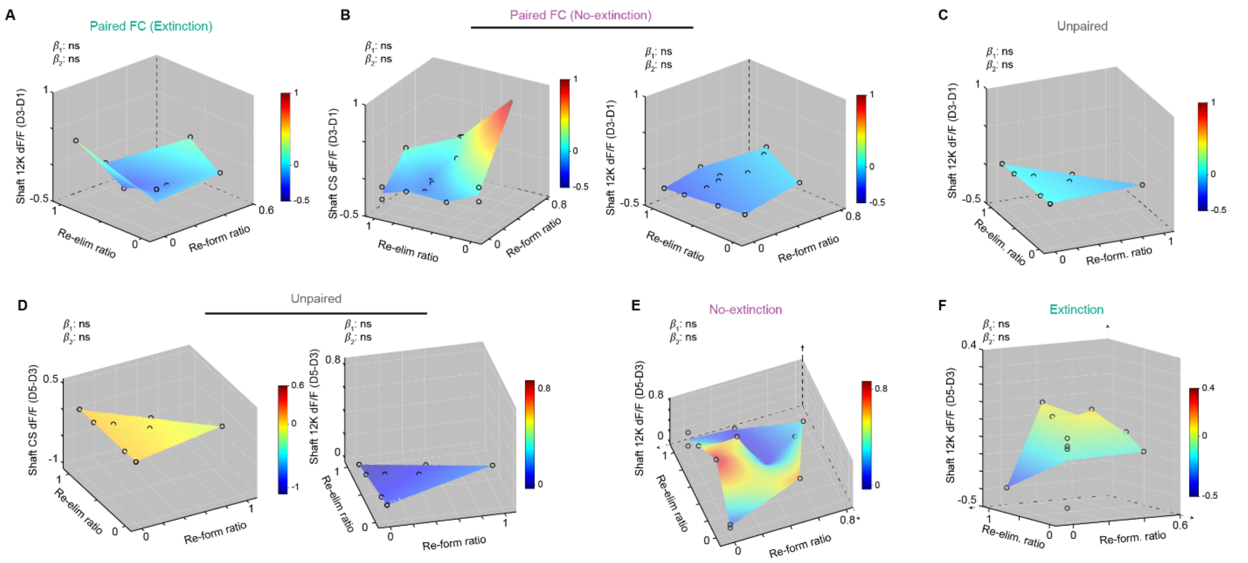

Fig. S7. ***Regression model between dendritic shaft activity and spine remodeling ratio.*** (**A** to **F**) Three-dimension scatter plot of dendrites in re-form ratio, re-elim ratio and shaft dF/F in response to CS or 12 kHz tone; β_1 denotes the coefficient for re-form ratio in the linear regression model; β_2 denotes the coefficient for re-elim ratio in the linear model; ns denotes no significant difference; linear mixed-effects model; n = 10 dendrites from 4 mice (A); n = 14 dendrites from 7 mice (B); n = 11 dendrites from 3 mice (C); n = 11 dendrites from 3 mice (D); n = 13 dendrites from 7 mice (E); n = 10 dendrites from 4 mice (F). Each circle represents a dendrite; the fitted surface is based on the values of all circles with the changing color showing the shaft CS dF/F.

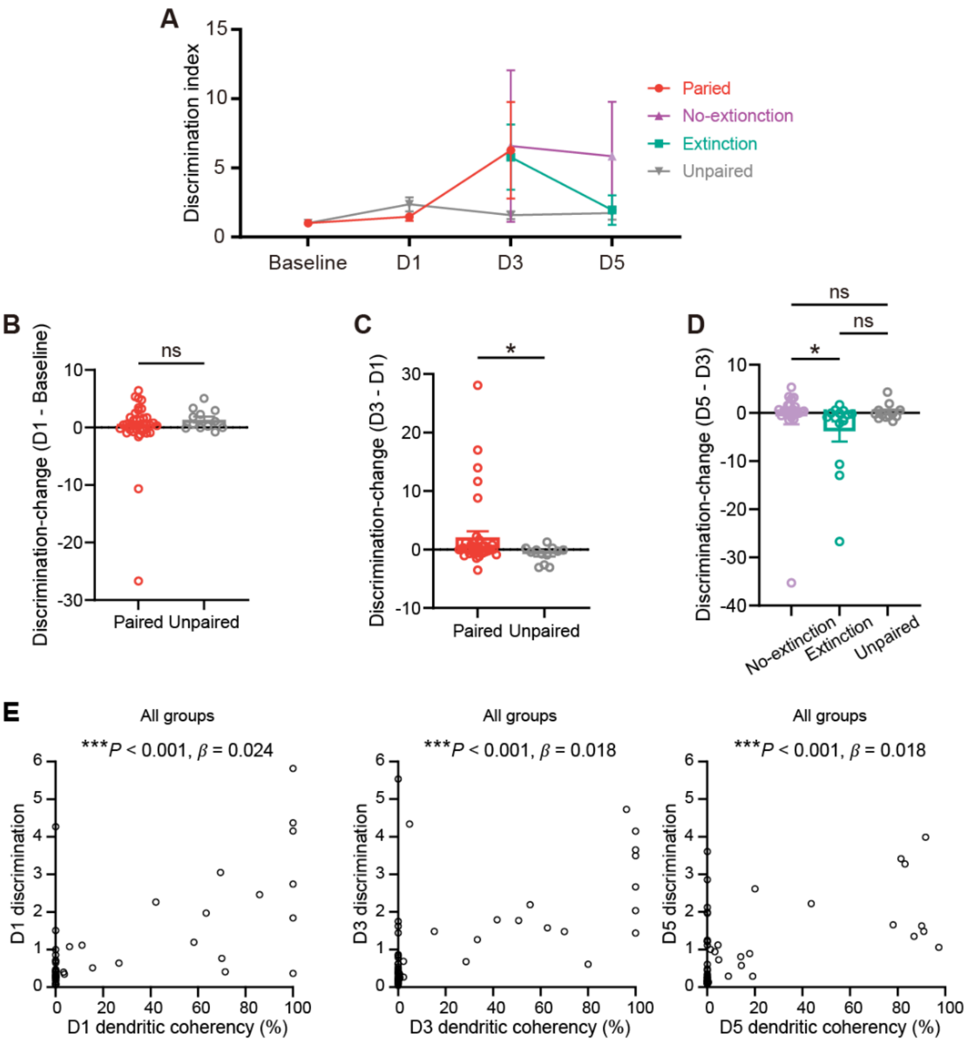

Fig. S8. ***Properties of dendritic discrimination.*** (**A**) Discrimination index of dendritic response at different sessions. Data shown as mean ± SEM. (**B** to **D**) Discrimination-change from one session to another. For (B) and (C), two-tailed Mann-Whitney test was used; for (D), Kruskal-Wallis test with *post hoc* Dunn's multiple comparisons test was used; $*P < 0.05$, ns: no significant difference. Data shown as mean ± SEM; paired, $n = 37$ dendrites from 11 mice; extinction, $n = 14$ dendrites from 4 mice; no-extinction, $n = 23$ dendrites from 7 mice; unpaired, $n = 12$ dendrites from 3 mice. (**E**) Relationship between dendritic discrimination index and dendritic coherency at Day 1, Day 3, and Day 5. Linear mixed-effects model (random effects: mouse-ID). P values and regression coefficients (β) are from the statistical model specified in Methods. Each point represents one dendrite; data is from all groups; $n = 49$ dendrites from 14 mice.

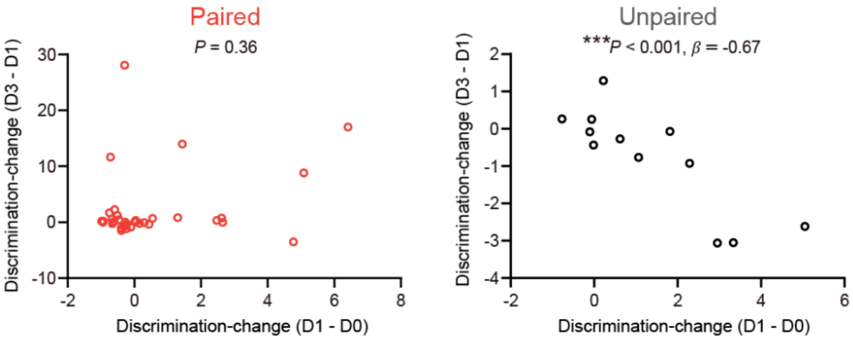

Fig. S9. ***Reversed dendritic discrimination change in unpaired group.*** Linear mixed-effects model testing the correlation between the change in discrimination index from D0 to D1 and discrimination-change from D1 to D3. Paired, *n* = 37 dendrites from 11 mice; unpaired, *n* = 12 dendrites from 3 mice.

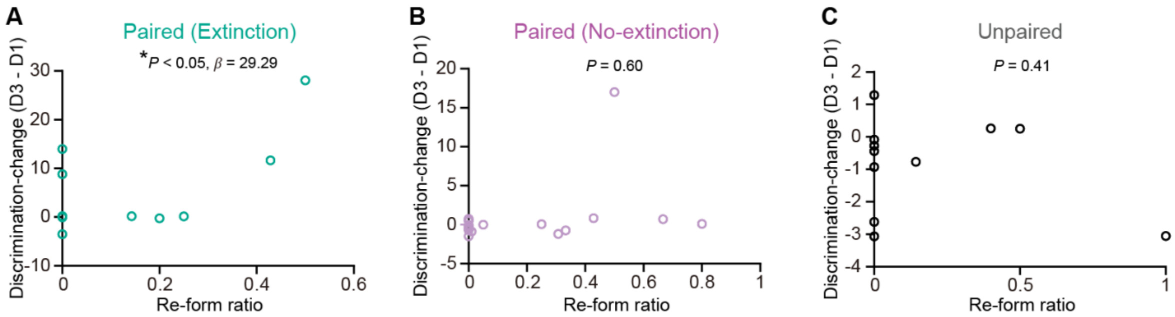

Fig. S10. ***Relationship between dendritic spine remodeling and discrimination change.*** (**A** to **C**) Linear mixed-effects model testing the correlation between the dendritic re-form ratio and discrimination-change from D1 to D3. Sample size in dendrite: extinction, *n* = 12 dendrites from 4 mice; no-extinction, *n* = 18 dendrites from 7 mice; unpaired, *n* = 11 dendrites from 3 mice.

Table S1.

Information of statistics in all figures. The table is in 6 columns to summarize the comparison groups with group size, main test, whether with post-host test or mixed effects, *P* value and other notes including R value, coefficients, homogeneity of variance test and normality test.

**Figure**	**Comparison groups (size)**	**Test**	**Post-hoc test/mixed effects**	***P* value**	**Others**
Fig. 1F: Baseline-4K	Pre vs CS: 623 spines from 49 dendrites from 14 mice	Linear mixed-effects model	Fixed effect: period (Pre or CS) Random intercepts: mouse, dendrite, spine	$P < 0.001$	
Fig. 1G: Baseline-12K	Pre vs CS: 623 spines from 49 dendrites from 14 mice	Linear mixed-effects model	Fixed effect: period (Pre or CS) Random intercepts: mouse, dendrite, spine	$P = 0.0489$	
Fig. 1H: D1-Paired-4K	Pre vs CS: 454 spines from 37 dendrites from 11 mice	Linear mixed-effects model	Fixed effect: period (Pre or CS) Random intercepts: mouse, dendrite, spine	$P < 0.001$	
Fig. 1I: D1-Unpaired-4K	Pre vs CS: 157 spines from 12 dendrites from 3 mice	Linear mixed-effects model	Fixed effect: period (Pre or CS) Random intercepts: mouse, dendrite, spine	$P < 0.0048$	
Fig. 1J: D3-Paired-4K	Pre vs CS: 430 spines from 37 dendrites from 11 mice	Linear mixed-effects model	Fixed effect: period (Pre or CS) Random intercepts: mouse, dendrite, spine	$P < 0.001$	
Fig. 1K: D3-Unpaired-4K	Pre vs CS: 144 spines from 12 dendrites from 3 mice	Linear mixed-effects model	Fixed effect: period (Pre or CS) Random intercepts: mouse, dendrite, spine	$P = 0.9155$	
Fig. 1L: D5-No extinction-4K	Pre vs CS: 27 5spines from 23 dendrites from 7 mice	Linear mixed-effects model	Fixed effect: period (Pre or CS) Random intercepts: mouse, dendrite, spine	$P = 0.3986$	
Fig. 1M: D5-Extinction-4K	Pre vs CS: 143 spines from 14 dendrites from 4 mice	Linear mixed-effects model	Fixed effect: period (Pre or CS) Random intercepts: mouse, dendrite, spine	$P = 0.1127$	
Fig. 1N: No extinction	D3 vs D5: 273 spines from 23 dendrites from 7 mice	Linear mixed-effects model with FDR correction	Fixed effect: D3 CS net-response Random intercepts: mouse, dendrite	$P = 0.0363$	$\beta = 0.2113$
Fig. 1O: Extinction	D3 vs D5: 141 spines from 14 dendrites from 4 mice	Linear mixed-effects model with FDR correction	Fixed effect: D3 CS net-response Random intercepts: mouse, dendrite	$P = 0.58995$	

Fig. 1P: Unpaired	D3 vs D5: 136 spines from 12 dendrites from 3 mice	Linear mixed-effects model with FDR correction	Fixed effect: D3 CS net-response Random intercepts: mouse, dendrite	P = 0.9479	
Fig. 1Q: No extinction	D3-D1 vs D5-D3: 216 spines from 23 dendrites from 7 mice	Linear mixed-effects model with FDR correction	Fixed effect: D1-D3 CS net-response Random intercepts: mouse, dendrite	P = 0.5614	
Fig. 1R: Extinction	D3-D1 vs D5-D3: 117 spines from 14 dendrites from 4 mice	Linear mixed-effects model with FDR correction	Fixed effect: D1-D3 CS net-response Random intercepts: mouse, dendrite	P < 0.0001	β = -0.4691
Fig. 1S: Unpaired	D3-D1 vs D5-D3: 119 spines from 12 dendrites from 3 mice	Linear mixed-effects model with FDR correction	Fixed effect: D1-D3 CS net-response Random intercepts: mouse, dendrite	P = 0.0039	β = -0.2877
Fig. 2B: D1_Paired	synchronous (451 pairs from 37 dendrites from 11 mice) vs Non-synchronous (6289 pairs from 37 dendrites from 11 mice)	Two-sample Kolmogorov-Smirnov test with FDR correction		P12 = 0.0678 P13 = 0.0782	
Fig. 2D: D1_Unpaired	synchronous (135 pairs from 12 dendrites from 3 mice) vs Non-synchronous (2046 pairs from 12 dendrites from 3 mice)	Two-sample Kolmogorov-Smirnov test with FDR correction		P12 = 0.3700 P13 = 0.1652	
Fig. 2F: D3_Paired	synchronous (489 pairs from 37 dendrites from 11 mice) vs Non-synchronous (6271 pairs from 37 dendrites from 11 mice)	Two-sample Kolmogorov-Smirnov test with FDR correction		P12 = 0.0062 P13 = 0.0062	

Fig. 2H: D3_Unpaired	synchronous (93 pairs from 12 dendrites from 3 mice) vs Non-synchronous (2088 pairs 12 dendrites from 3 mice)	Two-sample Kolmogorov-Smirnov test with FDR correction		P12 = 0.7889<br>P13 = 0.7889	
Fig. 2J: D5_No-Extinction	synchronous (204 pairs from 23 dendrites from 7 mice) vs Non-synchronous (4743 pairs from 23 dendrites from 7 mice)	Two-sample Kolmogorov-Smirnov test with FDR correction		P12 < 0.0001<br>P13 < 0.0001	
Fig. 2L: D5_Extinction	synchronous (123 pairs from 14 dendrites from 4 mice) vs Non-synchronous (1670 pairs from 14 dendrites from 4 mice)	Two-sample Kolmogorov-Smirnov test with FDR correction		P12 = 0.3085<br>P13 = 0.3085	
Fig. 2N: D5_unpair	synchronous (250 pairs from 12 dendrites from 3 mice) vs Non-synchronous (1931 pairs from 12 dendrites from 3 mice)	Two-sample Kolmogorov-Smirnov test with FDR correction		P12 = 0.0082<br>P13 = 0.0202	
Fig. 2C: D1_Paired	D1 vs synchronous: 454 spines from 37 dendrites from 11 mice	Linear mixed-effects model	Fixed effect: D1 CS net-response Random intercepts: mouse, dendrite	P = 0.0107	β = 0.0608
Fig. 2E: D1_Unpaired	D1 vs synchronous: 157 spines from 12 dendrites from 3 mice	Linear mixed-effects model	Fixed effect: D1 CS net-response Random intercepts: mouse, dendrite	P = 0.0829	

Fig. 2G: D3_Paired	D3 vs synchronous: 430 spines from 37 dendrites from 11 mice	Linear mixed-effects model	Fixed effect: D3 CS net-response Random intercepts: mouse, dendrite	P = 0.0012	β = 0.0744
Fig. 2I: D3_Unpaired	D3 vs synchronous: 144 spines from 12 dendrites from 3 mice	Linear mixed-effects model	Fixed effect: D3 CS net-response Random intercepts: mouse, dendrite	P = 0.4784	
Fig. 2K: D5_No-extinction	D5 vs synchronous: 275 spines from 23 dendrites from 7 mice	Linear mixed-effects model	Fixed effect: D5 CS net-response Random intercepts: mouse, dendrite	P = 0.0065	β = 0.0720
Fig. 2M: D5_Extinction	D5 vs synchronous: 143 spines from 14 dendrites from 4 mice	Linear mixed-effects model	Fixed effect: D5 CS net-response Random intercepts: mouse, dendrite	P = 0.3229	
Fig. 2O: D5_unpair	D5 vs synchronous: 137 spines from 12 dendrites from 3 mice	Linear mixed-effects model	Fixed effect: D5 CS net-response Random intercepts: mouse, dendrite	P = 0.0072	β = -0.1172
Fig. 2P: D1_dendrite	Paired: D1 vs synchronous: 37 dendrites from 11 mice	Linear mixed-effects model	Fixed effect: D1 CS dendritic net-response Random intercepts: mouse	P = 0.0023	β = 0.5362
	Unpaired: D1 vs synchronous: 12 dendrites from 3 mice	Linear mixed-effects model	Fixed effect: D1 CS dendritic net-response Random intercepts: mouse	P = 0.3396	
Fig. 2Q: D3_dendrite	Paired: D3 vs synchronous: 37 dendrites from 11 mice	Linear mixed-effects model	Fixed effect: D3 CS dendritic net-response Random intercepts: mouse	P < 0.0001	β = 0.6488
	Unpaired: D3 vs synchronous: 12 dendrites from 3 mice	Linear mixed-effects model	Fixed effect: D3 CS dendritic net-response Random intercepts: mouse	P = 0.0822	
Fig. 2R: D5_dendrite	No-extinction: D5 vs synchronous:	Linear mixed-effects model	Fixed effect: D5 CS dendritic net-response	P = 0.0008	β = 0.4816

	23 dendrites from 7 mice		Random intercepts: mouse		
Fig. 2Ss: D5_dendrite	Extinction: D5 vs synchronous: 14 dendrites from 4 mice	Linear mixed-effects model	Fixed effect: D5 CS dendritic net-response Random intercepts: mouse	P = 0.0134	β = 0.8186
Fig. 2T: D5_dendrite	Unpaired: D5 vs synchronous: 12 dendrites from 3 mice	Linear mixed-effects model	Fixed effect: D5 CS dendritic net-response Random intercepts: mouse	P = 0.0501	
Fig. 3E	Branch: 100	Paired t-test		P < 0.001	
	Local: 100	Paired t-test		P < 0.001	
Fig. 3F	Branch: 100	Paired t-test		P = 0.0094	
	Local: 100	Paired t-test		P < 0.001	
Fig. 3H	Branch: 80	Paired t-test		P = 0.7612	
Fig. 3I	Branch: 80	Paired t-test		P = 0.0018	
Fig. 3J	Branch: 80	Paired t-test		P = 0.0837	
Fig. 3K	Branch: 80	Paired t-test		P < 0.001	
Fig. 4C	$X_1 = a_{11}Z_1 + a_{12}Z_2$; $X_2 = a_{21}Z_1 + a_{22}Z_2$; $Y = b_1X_1 + b_2X_2$ n = 376 spines from 37 dendrites from 11 mice	Two-stage least squares regression	Stage1: Spine D1 ~ Spine Baseline + Neighbour Baseline; Neighbour D1 ~ Spine Baseline + Neighbour Baseline Stage2: Spine elimination ~ Spine D1 + Neighbour D1	Overall P = 0.0184; Stage1: P_{11} < 0.001; P_{12} < 0.001; P_{21} < 0.001; P_{22} < 0.001; Stage2: P_1 = 0.0046; P_2 = 0.0083	Stage1: a11 = 0.2205; a12 = 0.1104; a21 = -0.5060; a22 = 1.0805 Stage2: β1 = -6.8076; β2 = 0.9214
Fig. 4D	$X_1 = a_{11}Z_1 + a_{12}Z_2$; $X_2 = a_{21}Z_1 + a_{22}Z_2$; $Y = b_1X_1 + b_2X_2$ n = 128 spines from 12 dendrites from 3 mice	Two-stage least squares regression	Stage1: Spine D1 ~ Spine Baseline + Neighbour Baseline; Neighbour D1 ~ Spine Baseline + Neighbour Baseline Stage2: Spine elimination ~ Spine D1 + Neighbour D1	Overall P = 0.1211; Stage1: P_{11} = 0.9014; P_{12} = 0.1245; P_{21} < 0.001; P_{22} < 0.001; Stage2: P_1 =0.0913 ; P_2 = 0.0565	Stage1: a11 = 0.0119; a12 = -0.0713; a21 =-0.5331; a22 = 0.6397 Stage2: β1 = 66.0383; β2 = 8.2652
Fig. 5A: D1_D3e_Paired_4K	Pre vs CS: 105 spines from 37 dendrites from 11 mice	Linear mixed-effects model	Fixed effect: period (Pre or CS) Random intercepts: mouse, dendrite, spine	P = 0.4269	
Fig. 5A: D1_D3e_Paired_12K	Pre vs CS: 105 spines from 37 dendrites from 11 mice	Linear mixed-effects model	Fixed effect: period (Pre or CS) Random intercepts: mouse, dendrite, spine	P = 0.4256	

Fig. 5B: D1_D3s_Paired_4K	Pre vs CS: 349 spines from 37 dendrites from 11 mice	Linear mixed-effects model	Fixed effect: period (Pre or CS) Random intercepts: mouse, dendrite, spine	P < 0.001	
Fig. 5B: D1_D3s_Paired_12K	Pre vs CS: 349 spines from 37 dendrites from 11 mice	Linear mixed-effects model	Fixed effect: period (Pre or CS) Random intercepts: mouse, dendrite, spine	P = 0.4518	
Fig. 5C: D3_D3s_Paired_4K	Pre vs CS: 349 spines from 37 dendrites from 11 mice	Linear mixed-effects model	Fixed effect: period (Pre or CS) Random intercepts: mouse, dendrite, spine	P < 0.001	
Fig. 5C: D3_D3s_Paired_12K	Pre vs CS: 349 spines from 37 dendrites from 11 mice	Linear mixed-effects model	Fixed effect: period (Pre or CS) Random intercepts: mouse, dendrite, spine	P < 0.001	
Fig. 5D: D3_D3f_Paired_4K	Pre vs CS: 81 spines from 37 dendrites from 11 mice	Linear mixed-effects model	Fixed effect: period (Pre or CS) Random intercepts: mouse, dendrite, spine	P = 0.6661	
Fig. 5D: D3_D3f_Paired_12K	Pre vs CS: 81 spines from 37 dendrites from 11 mice	Linear mixed-effects model	Fixed effect: period (Pre or CS) Random intercepts: mouse, dendrite, spine	P = 0.1965	
Fig. 5E: D5_D3f_No-extinction_4K	Pre vs CS: 25 spines from 23 dendrites from 7 mice	Linear mixed-effects model	Fixed effect: period (Pre or CS) Random intercepts: mouse, dendrite, spine	P = 0.0390	
Fig. 5E: D5_D3f_No-extinction_12K	Pre vs CS: 25 spines from 23 dendrites from 7 mice	Linear mixed-effects model	Fixed effect: period (Pre or CS) Random intercepts: mouse, dendrite, spine	P = 0.0030	
Fig. 5E: D5_D3f_Extinction_4K	Pre vs CS: 12 spines from 14 dendrites from 4 mice	Linear mixed-effects model	Fixed effect: period (Pre or CS) Random intercepts: mouse, dendrite, spine	P = 0.3036	
Fig. 5E: D5_D3f_Extinction_12K	Pre vs CS: 12 spines from 14 dendrites from 4 mice	Linear mixed-effects model	Fixed effect: period (Pre or CS) Random intercepts: mouse, dendrite, spine	P = 0.3172	
Fig. 5F: D3e-D5f	Real: 16<br>Simulation: 25000	Two-sample Kolmogorov-Smirnov test		P < 0.001	
Fig. 5G: D3s-D5f	Real: 26			P = 0.7003	

	Simulation: 25000	Two-sample Kolmogorov-Smirnov test			
Fig. 5H: D3s-D3e	Real: 108<br>Simulation: 108000	Two-sample Kolmogorov-Smirnov test		P = 0.3883	
Fig. 5I: Extinction	D5f dF/F vs D3e-D5f: 25 spines from 14 dendrites from 4 mice	Linear mixed-effects model	Fixed effect: Distance Random intercepts: mouse	P = 0.0337	β = 0.0095
	D5f dF/F vs D3s-D5f: 25 spines from 14 dendrites from 4 mice	Linear mixed-effects model	Fixed effect: Distance Random intercepts: mouse	P = 0.2910	
	D5f dF/F vs D3s-D5e: 108 spines from 14 dendrites from 4 mice	Linear mixed-effects model	Fixed effect: Distance Random intercepts: mouse	P = 0.9234	
Fig. 5J: No-extinction	D5f dF/F vs D3e-D5f: 58 spines from 23 dendrites from 7 mice	Linear mixed-effects model	Fixed effect: Distance Random intercepts: mouse	P = 0.2655	
	D5f dF/F vs D3s-D5f: 58 spines from 23 dendrites from 7 mice	Linear mixed-effects model	Fixed effect: Distance Random intercepts: mouse	P = 0.3980	
	D5f dF/F vs D3s-D5e: 205 spines from 23 dendrites from 7 mice	Linear mixed-effects model	Fixed effect: Distance Random intercepts: mouse	P = 0.2325	
Fig. 6B: D5_D3e5f_No-extinction_4K	Pre vs CS: 22 spines from 23 dendrites from 7 mice	Linear mixed-effects model	Fixed effect: period (Pre or CS) Random intercepts: mouse, dendrite, spine	P = 0.9414	
Fig. 6B: D5_D3e5f_No-extinction_12K	Pre vs CS: 22 spines from 23 dendrites from 7 mice	Linear mixed-effects model	Fixed effect: period (Pre or CS) Random intercepts: mouse, dendrite, spine	P = 0.2461	
Fig. 6B: D5_new D5f_No-extinction_4K	Pre vs CS: 36 spines from 23 dendrites from 7 mice	Linear mixed-effects model	Fixed effect: period (Pre or CS) Random intercepts: mouse, dendrite, spine	P = 0.3664	
Fig. 6C: D5_D3e5f_Extinction_4K	Pre vs CS: 7 spines from 14	Linear mixed-effects model	Fixed effect: period (Pre or CS) Random intercepts:	P = 0.0251	

	dendrites from 4 mice		mouse, dendrite, spine		
Fig. 6C: D5_D3e5f_Extinction_12K	Pre vs CS: 7 spines from 14 dendrites from 4 mice	Linear mixed-effects model	Fixed effect: period (Pre or CS) Random intercepts: mouse, dendrite, spine	P = 0.9186	
Fig. 6C: D5_new D5f_Extinction_4K	Pre vs CS: 18 spines from 14 dendrites from 4 mice	Linear mixed-effects model	Fixed effect: period (Pre or CS) Random intercepts: mouse, dendrite, spine	P = 0.3769	
Fig. 6E: D3-D1_Paired(Extinction)_4K	Shaft vs Re-form: 10 dendrites from 4 mice	Linear mixed-effects model	Fixed effect: Shaft CS dF/F Random intercepts: mouse	P = 0.0038	β1 = 2.0443
	Shaft vs Re-elim: 10 dendrites from 4 mice	Linear mixed-effects model	Fixed effect: Shaft CS dF/F Random intercepts: mouse	P = 0.5354	
Fig. 6F: D3-D1_Unpaired_4K	Shaft vs Re-form: 11 dendrites from 3 mice	Linear mixed-effects model	Fixed effect: Shaft CS dF/F Random intercepts: mouse	P = 0.8323	
	Shaft vs Re-elim: 11 dendrites from 3 mice	Linear mixed-effects model	Fixed effect: Shaft CS dF/F Random intercepts: mouse	P = 0.5355	
Fig. 6G: D5-D3_Extinction_4K	Shaft vs Re-form: 10 dendrites from 4 mice	Linear mixed-effects model	Fixed effect: Shaft CS dF/F Random intercepts: mouse	P < 0.001	β1 = -2.0043
	Shaft vs Re-elim: 10 dendrites from 4 mice	Linear mixed-effects model	Fixed effect: Shaft CS dF/F Random intercepts: mouse	P = 0.3884	
Fig. 6H: D5-D3_No-extinction_4K	Shaft vs Re-form: 13 dendrites from 7 mice	Linear mixed-effects model	Fixed effect: Shaft CS dF/F Random intercepts: mouse	P = 0.2591	
	Shaft vs Re-elim: 13 dendrites from 7 mice	Linear mixed-effects model	Fixed effect: Shaft CS dF/F Random intercepts: mouse	P = 0.524	
Fig. 6L: Paired	D3 vs D0-D1: 37 dendrites from 11 mice	Linear mixed-effects model	Fixed effect: D3 discrimination Random intercepts: mouse	P = 0.0105	β = 0.4841
Fig. 6M: Unpaired	D3 vs D0-D1: 12 dendrites from 3 mice	Linear mixed-effects model	Fixed effect: D3 discrimination Random intercepts: mouse	P = 0.4764	

Fig. 6N: No-extinction	D3-D5 vs re-form: 18 dendrites from 7 mice	Linear mixed-effects model	Fixed effect: Shaft CS dF/F Random intercepts: mouse	P = 0.4284	
Fig. 6O: Extinction	D3-D5 vs re-form: 12 dendrites from 4 mice	Linear mixed-effects model	Fixed effect: D3 discrimination Random intercepts: mouse	P = 0.0105	β = -27.7579
Fig. 6P: Unpaired	D3-D5 vs re-form:12 dendrites from 3 mice	Linear mixed-effects model	Fixed effect: D3 discrimination Random intercepts: mouse	P = 0.7022	
Fig. S1C: Pearson's 15Hz	Synchronous (55) vs non-synchronous (11)	Unpaired t-test		P < 0.001	
Fig. S1C: FDR 15Hz	Synchronous (55) vs non-synchronous (11)	Unpaired t-test		P < 0.001	
Fig. S1C: Pearson's 1Hz	Synchronous (33) vs non-synchronous (33)	Unpaired t-test		P < 0.001	
Fig. S1C: FDR 1Hz	Synchronous (28) vs non-synchronous (38)	Unpaired t-test		P < 0.001	
Fig. S1D: Den#1 FDR 15Hz	Synchronous (26) vs non-synchronous (2)	Unpaired t-test		P = 0.702	
Fig. S1D: Den#1 FDR 1Hz	Synchronous (2) vs non-synchronous (26)	Unpaired t-test		P = 0.014	
Fig. S1D: Den#2 FDR 15Hz	Synchronous (66) vs non-synchronous (0)	Unpaired t-test		P = NA	
Fig. S1D: Den#2 FDR 1Hz	Synchronous (31) vs non-synchronous (35)	Unpaired t-test		P < 0.001	
Fig. S1D: Den#3 FDR 15Hz	Synchronous (55) vs non-synchronous (11)	Unpaired t-test		P < 0.001	
Fig. S1D: Den#3 FDR 1Hz	Synchronous (28) vs non-synchronous (38)	Unpaired t-test		P < 0.001	
Fig. S1E: Den#4 FDR 15Hz	Synchronous (55) vs non-	Unpaired t-test		P < 0.001	

	synchronous (11)				
Fig. S1E: Den#4 FDR 1Hz	Synchronous (28) vs non-synchronous (38)	Unpaired t-test		P < 0.001	
Fig. S1F: Den#5 FDR 15Hz	Synchronous (15) vs non-synchronous (13)	Unpaired t-test		P < 0.001	
Fig. S1F: Den#5 FDR 1Hz	Synchronous (2) vs non-synchronous (26)	Unpaired t-test		P = 0.011	
Fig. S1F: Den#6 FDR 15Hz	Synchronous (68) vs non-synchronous (23)	Unpaired t-test		P < 0.001	
Fig. S1F: Den#6 FDR 1Hz	Synchronous (26) vs non-synchronous (65)	Unpaired t-test		P < 0.001	
Fig. S2A: Baseline-4K	Pre vs CS: 623 spines from 49 dendrites from 14 mice	Linear mixed-effects model	Fixed effect: period (Pre or CS) Random intercepts: mouse, dendrite, spine	P < 0.001	
Fig. S2A: Baseline-12K	Pre vs CS: 623 spines from 49 dendrites from 14 mice	Linear mixed-effects model	Fixed effect: period (Pre or CS) Random intercepts: mouse, dendrite, spine	P = 0.0489	
Fig. S2A: D1-Paired-4K	Pre vs CS: 454 spines from 37 dendrites from 11 mice	Linear mixed-effects model	Fixed effect: period (Pre or CS) Random intercepts: mouse, dendrite, spine	P < 0.001	
Fig. S2A: D1-Paired-12K	Pre vs CS: 454 spines from 37 dendrites from 11 mice	Linear mixed-effects model	Fixed effect: period (Pre or CS) Random intercepts: mouse, dendrite, spine	P = 0.3247	
Fig. S2A: D1-Unpaired-4K	Pre vs CS: 157 spines from 12 dendrites from 3 mice	Linear mixed-effects model	Fixed effect: period (Pre or CS) Random intercepts: mouse, dendrite, spine	P < 0.0048	
Fig. S2A: D1-Unpaired-12K	Pre vs CS: 157 spines from 12 dendrites from 3 mice	Linear mixed-effects model	Fixed effect: period (Pre or CS) Random intercepts: mouse, dendrite, spine	P = 0.2636	

Fig. S2A: D3-Paired-4K	Pre vs CS: 430 spines from 37 dendrites from 11 mice	Linear mixed-effects model	Fixed effect: period (Pre or CS) Random intercepts: mouse, dendrite, spine	P < 0.001	
Fig. S2A: D3-Paired-12K	Pre vs CS: 430 spines from 37 dendrites from 11 mice	Linear mixed-effects model	Fixed effect: period (Pre or CS) Random intercepts: mouse, dendrite, spine	P < 0.001	
Fig. S2A: D3-Unpaired-4K	Pre vs CS: 144 spines from 12 dendrites from 3 mice	Linear mixed-effects model	Fixed effect: period (Pre or CS) Random intercepts: mouse, dendrite, spine	P < 0.9155	
Fig. S2A: D3-Unpaired-12K	Pre vs CS: 144 spines from 12 dendrites from 3 mice	Linear mixed-effects model	Fixed effect: period (Pre or CS) Random intercepts: mouse, dendrite, spine	P < 0.8024	
Fig. S2A: D5-No extinction-4K	Pre vs CS: 275 spines from 23 dendrites from 7 mice	Linear mixed-effects model	Fixed effect: period (Pre or CS) Random intercepts: mouse, dendrite, spine	P = 0.3986	
Fig. S2A: D5-No extinction-12K	Pre vs CS: 275 spines from 23 dendrites from 7 mice	Linear mixed-effects model	Fixed effect: period (Pre or CS) Random intercepts: mouse, dendrite, spine	P < 0.001	
Fig. S2A: D5-Extinction-4K	Pre vs CS: 143 spines from 14 dendrites from 4 mice	Linear mixed-effects model	Fixed effect: period (Pre or CS) Random intercepts: mouse, dendrite, spine	P = 0.1127	
Fig. S2A: D5-Extinction-12K	Pre vs CS: 143 spines from 14 dendrites from 4 mice	Linear mixed-effects model	Fixed effect: period (Pre or CS) Random intercepts: mouse, dendrite, spine	P = 0.9184	
Fig. S2A: D5-Unpaired-4K	Pre vs CS: spines from 12 dendrites from 3 mice	Linear mixed-effects model	Fixed effect: period (Pre or CS) Random intercepts: mouse, dendrite, spine	P < 0.001	
Fig. S2A: D5-Unpaired-12K	Pre vs CS: spines from 12 dendrites from 3 mice	Linear mixed-effects model	Fixed effect: period (Pre or CS) Random intercepts: mouse, dendrite, spine	P = 0.6291	
Fig. S2B: No-extinction Baseline	330 spines from 23 dendrites from 7 mice	Linear mixed-effects model	Fixed effect: period (Pre or CS) Random intercepts:	P < 0.001	

			mouse, dendrite, spine		
Fig. S2B: Extinction Baseline	173 spines from 144 dendrites from 4 mice	Linear mixed-effects model	Fixed effect: period (Pre or CS) Random intercepts: mouse, dendrite, spine	P < 0.001	
Fig. S2B: Unpaired Baseline	169 spines from 12 dendrites from 3 mice	Linear mixed-effects model	Fixed effect: period (Pre or CS) Random intercepts: mouse, dendrite, spine	P < 0.001	
Fig. S2C: Paired	D3 vs D5: 376 spines from 37 dendrites from 11 mice	Linear mixed-effects model	Fixed effect: Baseline CS net-response Random intercepts: mouse, dendrite	P = 0.1531	
Fig. S2D: Unpaired	D3 vs D5: 128 spines from 12 dendrites from 3 mice	Linear mixed-effects model	Fixed effect: Baseline CS net-response Random intercepts: mouse, dendrite	P = 0.3097	
Fig. S2E: Paired	D3 vs D5: 349 spines from 37 dendrites from 11 mice	Linear mixed-effects model	Fixed effect: D1 CS net-response Random intercepts: mouse, dendrite	P = 0.2267	
Fig. S2F: Unpaired	D3 vs D5: 114 spines from 12 dendrites from 3 mice	Linear mixed-effects model	Fixed effect: D1 CS net-response Random intercepts: mouse, dendrite	P = 0.0800	
Fig. S2G: Day 3	Unpaired (6) vs Paired (11)	Unpaired t-test		P < 0.001	Homogeneity of variance: Yes (P = 0.19) Normal distribution: Yes (P = 0.60)
Fig. S2G: Day 5	Unpaired (6) vs No-extinction (7) vs Extinction (4)	One-way ANOVA followed by Tukey's multiple comparisons test	ANOVA F (DFn, DFd) = 1.417 (2, 13)	Unpair vs. No extinction: P = 0.002; No extinction vs. Extinction: P = 0.001;	Normal distribution: Yes (P = 0.51)
Fig. S2H	Pre (9) vs tone (9)	Paired *t*-test		D1CS:0.003 D3CS:0.006 D5CS:0.403 D112k:0.827 D312k:0.201 D512k:0.946	

Fig. S2I	9	Pearson's correlation		P = 0.0436	R = 0.86
Fig. S3A: D1_Paired	D1 vs synchronous: 418 spines from 32 dendrites from 11 mice	Linear mixed-effects model	Fixed effect: D1 CS net-response Random intercepts: mouse, dendrite	P = 0.0047	β = 7.78
Fig. S3A: D1_Unpaired	D1 vs synchronous: 148 spines from 11 dendrites from 3 mice	Linear mixed-effects model	Fixed effect: D1 CS net-response Random intercepts: mouse, dendrite	P = 0.1591	
Fig. S3B: D3_Paired	D3 vs synchronous: 374 spines from 31 dendrites from 11 mice	Linear mixed-effects model	Fixed effect: D3 CS net-response Random intercepts: mouse, dendrite	P = 0.0070	β = 7.61
Fig. S3B: D3_Unpaired	D3 vs synchronous: 144 spines from 12 dendrites from 3 mice	Linear mixed-effects model	Fixed effect: D3 CS net-response Random intercepts: mouse, dendrite	P = 0.4784	
Fig. S3C: D5_No extinction	D5 vs synchronous: 270 spines from 23 dendrites from 7 mice	Linear mixed-effects model	Fixed effect: D5 CS net-response Random intercepts: mouse, dendrite	P = 0.0070	β = 7.05
Fig. S3C: D5_Extinction	D5 vs synchronous: 143 from 14 dendrites from 4 mice	Linear mixed-effects model	Fixed effect: D5 CS net-response Random intercepts: mouse, dendrite	P = 0.3229	
Fig. S3C: D5_Unpaired	D5 vs synchronous: 119 from 12 dendrites from 3 mice	Linear mixed-effects model	Fixed effect: D5 CS net-response Random intercepts: mouse, dendrite	P = 0.0017	β = -15.32
Fig. S3D: No-extinction	D1 (408 pairs) vs D3 (335 pairs) 23 dendrites from 7 mice	Two-sample Kolmogorov-Smirnov test with FDR correction		P = 0.8810	
Fig. S3B: D3_Paired	D3 (335 pairs) vs D5 (204 pairs) from 23 dendrites from 7 mice	Two-sample Kolmogorov-Smirnov test with FDR correction		P = 0.0073	

Fig. S3E: Extinction	D1 (43 pairs) vs D3 (134 pairs) from 14 dendrites from 4 mice	Two-sample Kolmogorov-Smirnov test with FDR correction		P = 0.8342	
Fig. S3C: D5_No extinction	D3 (134 pairs) vs D5 (123 pairs) from 14 dendrites from 4 mice	Two-sample Kolmogorov-Smirnov test with FDR correction		P = 0.2765	
Fig. S3F: Unpaired	D1 (135 pairs) vs D3 (93 pairs) from 12 dendrites from 3 mice	Two-sample Kolmogorov-Smirnov test with FDR correction		P = 0.8221	
Fig. S3C: D5_Unpaired	D3 (93 pairs) vs D5 (250 pairs) from 12 dendrites from 3 mice	Two-sample Kolmogorov-Smirnov test with FDR correction		P = 0.2215	
Fig. S3L: D1<br>Fig. S3L: D3	synchronous (16744) vs Non-synchronous (2626)	Linear mixed-effects model	Fixed effect: spine type (synchronous or Non-synchronous)<br>Random intercepts: dendrite	P < 0.001	
Fig. S3F: Unpaired	synchronous (14934) vs Non-synchronous (1019)	Linear mixed-effects model	Fixed effect: spine type (synchronous or Non-synchronous)<br>Random intercepts: dendrite	P < 0.001	
Fig. S4A<br>Fig. S4B	Elim vs Spine dF/F: 128 spines from 37 dendrites from 11 mice	Logistic model		P = 0.0279	β = -3.2086
Fig. S3L: D1<br>Fig. S3L: D3<br>Fig. S5A: D1_D3e_Unpaired_4K<br>Fig. S5A: D1_D3e_Unpaired_12K	Elim vs Neighbour dF/F: 128 spines from 37 dendrites from 11 mice	Logistic model		P = 0.6720	
	Pre vs CS: 43 spines from 12 dendrites from 3 mice	Linear mixed-effects model	Fixed effect: period (Pre or CS)<br>Random intercepts: mouse, dendrite, spine	P = 0.2001	
Fig. S4A<br>Fig. S4B<br>Fig. S5B: D1_D3s_Unpaired_4K	Pre vs CS: 43 spines from 12 dendrites from 3 mice	Linear mixed-effects model	Fixed effect: period (Pre or CS)<br>Random intercepts: mouse, dendrite, spine	P = 0.6263	

	Pre vs CS: 114 spines from 12 dendrites from 3 mice	Linear mixed-effects model	Fixed effect: period (Pre or CS) Random intercepts: mouse, dendrite, spine	P = 0.0072	
Fig. S5B: D1_D3s_Unpaired_12K Fig. S5C: D3_D3s_Unpaired_4K	Pre vs CS: 114 spines from 12 dendrites from 3 mice	Linear mixed-effects model	Fixed effect: period (Pre or CS) Random intercepts: mouse, dendrite, spine	P = 0.1512	
	Pre vs CS: 114 spines from 12 dendrites from 3 mice	Linear mixed-effects model	Fixed effect: period (Pre or CS) Random intercepts: mouse, dendrite, spine	P = 0.4402	
Fig. S5C: D3_D3s_Unpaired_12K	Pre vs CS: 114 spines from 12 dendrites from 3 mice	Linear mixed-effects model	Fixed effect: period (Pre or CS) Random intercepts: mouse, dendrite, spine	P = 0.6639	
Fig. S5D: D3_D3f_Unpaired_4K	Pre vs CS: 30 spines from 12 dendrites from 3 mice	Linear mixed-effects model	Fixed effect: period (Pre or CS) Random intercepts: mouse, dendrite, spine	P = 0.0138	
Fig. S5D: D3_D3f_Unpaired_12K	Pre vs CS: 30 spines from 12 dendrites from 3 mice	Linear mixed-effects model	Fixed effect: period (Pre or CS) Random intercepts: mouse, dendrite, spine	P = 0.0811	
Fig. S6A: D5_D3e5f_Unpaired_4K	Pre vs CS: 5 spines from 12 dendrites from 3 mice	Linear mixed-effects model	Fixed effect: period (Pre or CS) Random intercepts: mouse, dendrite, spine	P = 0.0857	
Fig.S6A: D5_D3e5f_Unpaired_12K	Pre vs CS: 5 spines from 12 dendrites from 3 mice	Linear mixed-effects model	Fixed effect: period (Pre or CS) Random intercepts: mouse, dendrite, spine	P = 0.6410	
Fig. S6A: D5_new D5f_Unpaired_4K	Pre vs CS: 12 spines from 12 dendrites from 3 mice	Linear mixed-effects model	Fixed effect: period (Pre or CS) Random intercepts: mouse, dendrite, spine	P = 0.7589	
Fig. S6B: 4K	Pre vs CS: 4 spines from 14 dendrites from 4 mice	Linear mixed-effects model	Fixed effect: period (Pre or CS) Random intercepts: mouse, dendrite, spine	P = 0.0039	
Fig. S6B: 12K	Pre vs 12K: 4 spines from 14 dendrites from 4 mice	Linear mixed-effects model	Fixed effect: period (Pre or CS) Random intercepts:	P = 0.2227	

			mouse, dendrite, spine		
Fig. S6C: D3_D3f5e_No-extinction_4K	Pre vs CS: 36 spines from 23 dendrites from 7 mice	Linear mixed-effects model	Fixed effect: period (Pre or CS) Random intercepts: mouse, dendrite, spine	P = 0.8811	
Fig. S6C: D3_D3f5e_No-extinction_12K	Pre vs 12K: 36 spines from 23 dendrites from 7 mice	Linear mixed-effects model	Fixed effect: period (Pre or CS) Random intercepts: mouse, dendrite, spine	P = 0.0600	
Fig. S6C: D3_D3f5e_Extinction_4K	Pre vs CS: 8 spines from 14 dendrites from 4 mice	Linear mixed-effects model	Fixed effect: period (Pre or CS) Random intercepts: mouse, dendrite, spine	P = 0.4025	
Fig. S6C: D3_D3f5e_Extinction_12K	Pre vs 12K: 8 spines from 14 dendrites from 4 mice	Linear mixed-effects model	Fixed effect: period (Pre or CS) Random intercepts: mouse, dendrite, spine	P = 0.1588	
Fig. S6C: D3_D3f5e_Unpaired_4K	Pre vs CS: 15 spines from 12 dendrites from 3 mice	Linear mixed-effects model	Fixed effect: period (Pre or CS) Random intercepts: mouse, dendrite, spine	P = 0.0943	
Fig. S6C: D3_D3f5e_Unpaired_12K	Pre vs 12K: 15 spines from 2 dendrites from 3 mice	Linear mixed-effects model	Fixed effect: period (Pre or CS) Random intercepts: mouse, dendrite, spine	P = 0.3030	
Fig. S7A: D3-D1_Paired(Extinction)_12K	Shaft vs Re-form: 10 dendrites from 4 mice	Linear mixed-effects model	Fixed effect: Shaft CS dF/F Random intercepts: mouse	P = 0.5850	
Fig. S6C: D3_D3f5e_No-extinction_12K	Shaft vs Re-elim: 10 dendrites from 4 mice	Linear mixed-effects model	Fixed effect: Shaft CS dF/F Random intercepts: mouse	P = 0.5252	
Fig. S7B: D3-D1_Paired(No-extinction)_4K	Shaft vs Re-form: 14 dendrites from 7 mice	Linear mixed-effects model	Fixed effect: Shaft CS dF/F Random intercepts: mouse	P = 0.0941	
Fig. S6C: D3_D3f5e_Extinction_12K	Shaft vs Re-elim: 14 dendrites from 7 mice	Linear mixed-effects model	Fixed effect: Shaft CS dF/F Random intercepts: mouse	P = 0.4763	
Fig. S7B: D3-D1_Paired(No-extinction)_12K	Shaft vs Re-form: 14 dendrites from 7 mice	Linear mixed-effects model	Fixed effect: Shaft CS dF/F Random intercepts: mouse	P = 0.2129	

Fig. S6C: D3_D3f5e_Unpaired_12K	Shaft vs Re-elim: 14 dendrites from 7 mice	Linear mixed-effects model	Fixed effect: Shaft CS dF/F Random intercepts: mouse	P = 0.8418	
Fig. S7C: D3-D1_Unpaired_12K	Shaft vs Re-form: 11 dendrites from 3 mice	Linear mixed-effects model	Fixed effect: Shaft CS dF/F Random intercepts: mouse	P = 0.2595	
Fig. S7B: D3-D1_Paired(No-extinction)_12K	Shaft vs Re-elim: 11 dendrites from 3 mice	Linear mixed-effects model	Fixed effect: Shaft CS dF/F Random intercepts: mouse	P = 0.8462	
Fig. S7D: D5-D3_Unpaired_4K	Shaft vs Re-form: 11 dendrites from 3 mice	Linear mixed-effects model	Fixed effect: Shaft CS dF/F Random intercepts: mouse	P = 0.2741	
Fig. S7C: D3-D1_Unpaired_12K Fig. S7D: D5-D3_Unpaired_12K	Shaft vs Re-elim: 11 dendrites from 3 mice	Linear mixed-effects model	Fixed effect: Shaft CS dF/F Random intercepts: mouse	P = 0.9798	
	Shaft vs Re-form: 11 dendrites from 3 mice	Linear mixed-effects model	Fixed effect: Shaft CS dF/F Random intercepts: mouse	P = 0.5362	
Fig. S7D: D5-D3_Unpaired_4K Fig. S7E: D5-D3_No-extinction_12K	Shaft vs Re-elim: 11 dendrites from 3 mice	Linear mixed-effects model	Fixed effect: Shaft CS dF/F Random intercepts: mouse	P = 0.3825	
	Shaft vs Re-form: 13 dendrites from 7 mice	Linear mixed-effects model	Fixed effect: Shaft CS dF/F Random intercepts: mouse	P = 0.7728	
Fig. S7D: D5-D3_Unpaired_12K Fig. S7F: D5-D3_Extinction_12K	Shaft vs Re-elim: 13 dendrites from 7 mice	Linear mixed-effects model	Fixed effect: Shaft CS dF/F Random intercepts: mouse	P = 0.0696	
	Shaft vs Re-form: 10 dendrites from 4 mice	Linear mixed-effects model	Fixed effect: Shaft CS dF/F Random intercepts: mouse	P = 0.0748	Homogeneity of variance: No (P < 0.001) Normal distribution: No (P < 0.001)
Fig. S7E: D5-D3_No-extinction_12K Fig. S8B: D1-D0 Fig. S8C: D3-D1	Shaft vs Re-elim: 10 dendrites from 4 mice	Linear mixed-effects model	Fixed effect: Shaft CS dF/F Random intercepts: mouse	P = 0.9148	Homogeneity of variance: No (P < 0.001) Normal distribution: No (P < 0.001)
	Paired (37 dendires from	Mann-Whitney test		P = 0.4275	Homogeneity of variance:

	11 mice) vs Unpaired (12 dendrites from 3 mice)				Yes (P = 0.4758) Normal distribution: No (P < 0.001)
Fig. S7F: D5-D3_Extinction_12K<br>Fig. S8D: D5-D3<br>Fig. S8E: D1	Paired (37 dendires from 11 mice) vs Unpaired (12 dendrites from 3 mice)	Mann-Whitney test		P = 0.0284	
	No-extinction (14 dendrites from 4 mice) vs Extinction (23 dendrites from 7 mice) vs Unpaired (12 dendritres from 3 mice)	Kruskal-Wallis test	Dunn's multiple comparisons test	No-extinction vs Extinction: P = 0.0450; No-extinction vs Unpaired: P = 0.8574; Paired vs Unpaired: P = 0.7673	β = -0.6727
Fig. S8B: D1-D0<br>Fig. S8C: D3-D1<br>Fig. S8E: D3<br>Fig. S8E: D5	Dendritic coherency vs discrimination: 49 dendrites from 14 mice	Linear mixed-effects model	Fixed effect: Discrimination-change Random intercepts: mouse	P < 0.001	β = 0.024
	Dendritic coherency vs discrimination: 49 dendrites from 14 mice	Linear mixed-effects model	Fixed effect: Discrimination-change Random intercepts: mouse	P < 0.001	β = 0.018
Fig. S8D: D5-D3<br>Fig. S8E: D1<br>Fig. S9: Paired	Dendritic coherency vs discrimination: 49 dendrites from 14 mice	Linear mixed-effects model	Fixed effect: Discrimination-change Random intercepts: mouse	P < 0.001	β = 0.018
	D1-D3 vs D0-D1: 37 dendrites from 11 mice	Linear mixed-effects model	Fixed effect: Discrimination-change Random intercepts: mouse	P = 0.3561	
Fig. S9: Unpaired<br>Fig. S10A: Paired (Extinction)	D1-D3 vs D0-D1: 12 dendries from 3 mice	Linear mixed-effects model	Fixed effect: Discrimination-change Random intercepts: mouse	P = 0.0003	β = -0.6727
	D1-D3 vs re-form: 12 dendrites from 4 mice	Linear mixed-effects model	Fixed effect: Discrimination-change Random intercepts: mouse	P = 0.0149	

Fig. S10B: Paired (No-xtinction)	D1-D3 vs re-form: 18 dendrites from 7 mice	Linear mixed-effects model	Fixed effect: Discrimination-change Random intercepts: mouse	P = 0.5991	
Fig. S10C: Unpaired	D1-D3 vs re-form: 11 dendrites from 3 mice	Linear mixed-effects model	Fixed effect: Discrimination-change Random intercepts: mouse	P = 0.4101	

Table S2.

Sensitivity analysis of model outcomes across parameter settings. Parameter values were varied around the baseline configuration (100%) by scaling the HSP time constant (80–120%), the STDP weight-update magnitude (80–120%), and the STDP time constant (80–120%). The upper panel reports the dendritic local segment mean AUC (mV^2) for synchronous versus non-synchronous spine-pair stimulation on Day 1 (D1) and Day 3 (D3) for each parameter set. The lower panel reports the number of tuned dendrites in the weight-restoration simulations under "normal" vs "elimination restoration" conditions, categorized as CS-tuned dendrites (response of CS significantly > 12-kHz tone), 12-kHz-tuned dendrites (response of 12-kHz tone significantly > CS), or non-tuned (NA) dendrites.

Dendritic local segment mean AUC (mV^2) in spine pair stimulation

	D1			D3	
Parameters / Stimulation type	Synchronous stimulation	Non-synchronous stimulation		Synchronous stimulation	Non-synchronous stimulation
100%Parameters	3320.16362	2736.87696		4219.8946	3889.94905
HSP_80%τ	2983.7899	1949.06419		3467.88377	3366.54432
HSP_90%τ	3009.83319	2726.15312		4076.808	3847.59651
HSP_110%τ	2730.72565	2795.81426		3525.55646	3814.02043
HSP_120%τ	2918.3636	1798.85556		3376.31666	3212.01582
STDP_80%weight	2987.74284	2758.0202		2819.32138	2572.82799
STDP_90%weight	2590.89455	2625.66534		4756.00033	4187.13657
STDP_110%weight	4317.04815	3921.75924		3842.16116	3553.24964
STDP_120%weight	4350.47409	3420.78206		3963.33407	3399.60014
STDP_80%τ	2434.18681	2327.95947		3332.48094	2639.15538
STDP_90%τ	2192.37533	1400.80146		1919.46528	1898.86063
STDP_110%τ	3403.63271	2712.27624		3756.08734	2525.27451
STDP_120%τ	3892.19202	3293.15758		4149.58805	3752.00319

Number of tuning dendrites in weight restoration

	Normal				Elimination restoration		
Parameters / Tuning dendrite	CS-dendrite	12 kHz-dendrite	NA dendrite		CS-dendrite	12 kHz-dendrite	NA dendrite
100%Parameters	45	17	18		30	42	8
HSP_80%τ	45	17	18		30	42	8
HSP_90%τ	45	17	18		30	42	8
HSP_110%τ	45	17	18		30	42	8
HSP_120%τ	45	17	18		30	42	8
STDP_80%weight	59	7	14		47	22	11
STDP_90%weight	44	30	6		23	47	10
STDP_110%weight	44	28	8		31	37	12
STDP_120%weight	58	13	9		16	57	7
STDP_80%τ	79	1	0		36	35	9
STDP_90%τ	60	16	4		21	50	9
STDP_110%τ	53	18	8		6	69	5
STDP_120%τ	58	11	11		6	69	5

REFERENCES

1. G. Buzsaki, Neural syntax: Cell assemblies, synapsembles, and readers. *Neuron* **68**, 362–385 (2010).

2. B. A. Bicknell, M. Hausser, A synaptic learning rule for exploiting nonlinear dendritic computation. *Neuron* **109**, 4001–4017.e10 (2021).

3. G. Yang, F. Pan, W. B. Gan, Stably maintained dendritic spines are associated with lifelong memories. *Nature* **462**, 920–924 (2009).

4. M. Fisek, D. Herrmann, A. Egea-Weiss, M. Cloves, L. Bauer, T. Y. Lee, L. E. Russell, M. Hausser, Cortico-cortical feedback engages active dendrites in visual cortex. *Nature* **617**, 769–776 (2023).

5. H. Markram, J. Lubke, M. Frotscher, B. Sakmann, Regulation of synaptic efficacy by coincidence of postsynaptic APs and EPSPs. *Science* **275**, 213–215 (1997).

6. R. C. Malenka, R. A. Nicoll, NMDA-receptor-dependent synaptic plasticity: Multiple forms and mechanisms. *Trends Neurosci.* **16**, 521–527 (1993).

7. Y. Yang, D. Q. Liu, W. Huang, J. Deng, Y. Sun, Y. Zuo, M. M. Poo, Selective synaptic remodeling of amygdalocortical connections associated with fear memory. *Nat. Neurosci.* **19**, 1348–1355 (2016).

8. S. A. Josselyn, S. Tonegawa, Memory engrams: Recalling the past and imagining the future. *Science* **367**, eaaw4325 (2020).

9. J. H. Choi, S. E. Sim, J. I. Kim, D. I. Choi, J. Oh, S. Ye, J. Lee, T. Kim, H. G. Ko, C. S. Lim, B. K. Kaang, Interregional synaptic maps among engram cells underlie memory formation. *Science* **360**, 430–435 (2018).

10. K. Abdou, M. Shehata, K. Choko, H. Nishizono, M. Matsuo, S. I. Muramatsu, K. Inokuchi, Synapse-specific representation of the identity of overlapping memory engrams. *Science* **360**, 1227–1231 (2018).

11. K. Powell, A. Mathy, I. Duguid, M. Hausser, Synaptic representation of locomotion in single cerebellar granule cells. *eLife* **4**, e07290 (2015).

12. C. S. Lai, T. F. Franke, W. B. Gan, Opposite effects of fear conditioning and extinction on dendritic spine remodelling. *Nature* **483**, 87–91 (2012).

13. C. S. W. Lai, A. Adler, W. B. Gan, Fear extinction reverses dendritic spine formation induced by fear conditioning in the mouse auditory cortex. *Proc. Natl. Acad. Sci. U.S.A.* **115**, 9306–9311 (2018).

14. J. Nishiyama, R. Yasuda, Biochemical computation for spine structural plasticity. *Neuron* **87**, 63–75 (2015).

15. G. Yang, C. S. Lai, J. Cichon, L. Ma, W. Li, W. B. Gan, Sleep promotes branch-specific formation of dendritic spines after learning. *Science* **344**, 1173–1178 (2014).

16. D. I. Choi, J. Kim, H. Lee, J. I. Kim, Y. Sung, J. E. Choi, S. J. Venkat, P. Park, H. Jung, B. K. Kaang, Synaptic correlates of associative fear memory in the lateral amygdala. *Neuron* **109**, 2717–2726.e3 (2021).

17. S. Zhang, M. Xu, W. C. Chang, C. Ma, J. P. Hoang Do, D. Jeong, T. Lei, J. L. Fan, Y. Dan, Organization of long-range inputs and outputs of frontal cortex for top-down control. *Nat. Neurosci.* **19**, 1733–1742 (2016).

18. L. Topolnik, O. Camire, Non-linear calcium signalling and synaptic plasticity in interneurons. *Curr. Opin. Neurobiol.* **54**, 98–103 (2019).

19. P. Poirazi, B. W. Mel, Impact of active dendrites and structural plasticity on the memory capacity of neural tissue. *Neuron* **29**, 779–796 (2001).

20. K. A. Archie, B. W. Mel, A model for intradendritic computation of binocular disparity. *Nat. Neurosci.* **3**, 54–63 (2000).

21. K. F. Lee, C. Soares, J. P. Thivierge, J. C. Beique, Correlated synaptic inputs drive dendritic calcium amplification and cooperative plasticity during clustered synapse development. *Neuron* **89**, 784–799 (2016).

22. S. L. Smith, I. T. Smith, T. Branco, M. Hausser, Dendritic spikes enhance stimulus selectivity in cortical neurons in vivo. *Nature* **503**, 115–120 (2013).

23. M. London, M. Hausser, Dendritic computation. *Annu. Rev. Neurosci.* **28**, 503–532 (2005).

24. J. Schiller, G. Major, H. J. Koester, Y. Schiller, NMDA spikes in basal dendrites of cortical pyramidal neurons. *Nature* **404**, 285–289 (2000).

25. Q. Qiao, C. L. Wu, L. Ma, H. Zhang, M. Li, X. J. Wu, W. B. Gan, Motor learning-induced new dendritic spines are preferentially involved in the learned task than existing spines. *Cell Rep.* **40**, 111229 (2022).

26. W. Li, L. Ma, G. Yang, W. B. Gan, REM sleep selectively prunes and maintains new synapses in development and learning. *Nat. Neurosci.* **20**, 427–437 (2017).

27. W. J. Wright, N. G. Hedrick, T. Komiyama, Distinct synaptic plasticity rules operate across dendritic compartments in vivo during learning. *Science* **388**, 322–328 (2025).

28. A. Kerlin, B. Mohar, D. Flickinger, B. J. MacLennan, M. B. Dean, C. Davis, N. Spruston, K. Svoboda, Functional clustering of dendritic activity during decision-making. *eLife* **8**, e46966 (2019).

29. M. Sheng, D. Lu, R. H. Roth, F. J. Hwang, K. Sheng, J. B. Ding, Remodelling of corticostriatal axonal boutons during motor learning. *Nature* **646**, 143–151 (2025).

30. D. Nakayama, Z. Baraki, K. Onoue, Y. Ikegaya, N. Matsuki, H. Nomura, Frontal association cortex is engaged in stimulus integration during associative learning. *Curr. Biol.* **25**, 117–123 (2015).

31. M. Aime, E. Augusto, V. Kouskoff, T. Campelo, C. Martin, Y. Humeau, N. Chenouard, F. Gambino, The integration of Gaussian noise by long-range amygdala inputs in frontal circuit promotes fear learning in mice. *eLife* **9**, e62594 (2020).

32. M. F. Iacaruso, I. T. Gasler, S. B. Hofer, Synaptic organization of visual space in primary visual cortex. *Nature* **547**, 449–452 (2017).

33. P. J. Sjostrom, E. A. Rancz, A. Roth, M. Hausser, Dendritic excitability and synaptic plasticity. *Physiol. Rev.* **88**, 769–840 (2008).

34. D. N. Hill, Z. Varga, H. Jia, B. Sakmann, A. Konnerth, Multibranch activity in basal and tuft dendrites during firing of layer 5 cortical neurons in vivo. *Proc. Natl. Acad. Sci. U.S.A.* **110**, 13618–13623 (2013).

35. N. G. Hedrick, W. J. Wright, T. Komiyama, Local and global predictors of synapse elimination during motor learning. *Sci. Adv.* **10**, eadk0540 (2024).

36. M. T. Harnett, J. K. Makara, N. Spruston, W. L. Kath, J. C. Magee, Synaptic amplification by dendritic spines enhances input cooperativity. *Nature* **491**, 599–602 (2012).

37. B. L. Bloodgood, B. L. Sabatini, Nonlinear regulation of unitary synaptic signals by $CaV_{2.3}$ voltage-sensitive calcium channels located in dendritic spines. *Neuron* **53**, 249–260 (2007).

38. G. Kastellakis, D. J. Cai, S. C. Mednick, A. J. Silva, P. Poirazi, Synaptic clustering within dendrites: An emerging theory of memory formation. *Prog. Neurobiol.* **126**, 19–35 (2015).

39. S. Dura-Bernal, S. A. Neymotin, B. A. Suter, J. Dacre, J. V. S. Moreira, E. Urdapilleta, J. Schiemann, I. Duguid, G. M. G. Shepherd, W. W. Lytton, Multiscale model of primary motor cortex circuits predicts in vivo cell-type-specific, behavioral state-dependent dynamics. *Cell Rep.* **42**, 112574 (2023).

40. S. Dura-Bernal, E. Y. Griffith, A. Barczak, M. N. O'Connell, T. McGinnis, J. V. S. Moreira, C. E. Schroeder, W. W. Lytton, P. Lakatos, S. A. Neymotin, Data-driven multiscale model of

macaque auditory thalamocortical circuits reproduces in vivo dynamics. *Cell Rep.* **42**, 113378 (2023).

41. D. F. Tome, Y. Zhang, T. Aida, O. Mosto, Y. Lu, M. Chen, S. Sadeh, D. S. Roy, C. Clopath, Dynamic and selective engrams emerge with memory consolidation. *Nat. Neurosci.* **27**, 561–572 (2024).

42. S. d'Aquin, A. Szonyi, M. Mahn, S. Krabbe, J. Grundemann, A. Luthi, Compartmentalized dendritic plasticity during associative learning. *Science* **376**, eabf7052 (2022).

43. Q. Zheng, Y. Huang, C. Mu, X. Hu, C. S. W. Lai, Selective modulation of fear memory in non-rapid eye movement sleep. *Adv. Sci. (Weinh)* **11**, e2400662 (2024).

44. A. Goto, A. Bota, K. Miya, J. Wang, S. Tsukamoto, X. Jiang, D. Hirai, M. Murayama, T. Matsuda, T. J. McHugh, T. Nagai, Y. Hayashi, Stepwise synaptic plasticity events drive the early phase of memory consolidation. *Science* **374**, 857–863 (2021).

45. I. S. Stein, K. Zito, Dendritic spine elimination: Molecular mechanisms and implications. *Neuroscientist* **25**, 27–47 (2019).

46. I. E. Marinescu, P. N. Lawlor, K. P. Kording, Quasi-experimental causality in neuroscience and behavioural research. *Nat. Hum. Behav.* **2**, 891–898 (2018).

47. D. H. Bailey, A. J. Jung, A. M. Beltz, M. I. Eronen, C. Gische, E. L. Hamaker, K. P. Kording, C. Lebel, M. A. Lindquist, J. Moeller, A. Razi, J. M. Rohrer, B. Zhang, K. Murayama, Causal inference on human behaviour. *Nat. Hum. Behav.* **8**, 1448–1459 (2024).

48. B. Taschler, S. M. Smith, T. E. Nichols, Causal inference on neuroimaging data with Mendelian randomisation. *Neuroimage* **258**, 119385 (2022).

49. T. Liu, L. Ungar, K. Kording, Quantifying causality in data science with quasi-experiments. *Nat. Comput. Sci.* **1**, 24–32 (2021).

50. S. Hiu, T. Yong, J. Hasoon, M. D. Teare, J. P. Taylor, N. Lin, Instrumental variables in real-world clinical studies of dementia and neurodegenerative disease: Systematic review of the subject-matter argumentation, falsification test, and study design strategies to justify a valid instrument. *Brain Behav.* **14**, e3371 (2024).

51. M. E. Lepperod, T. Stober, T. Hafting, M. Fyhn, K. P. Kording, Inferring causal connectivity from pairwise recordings and optogenetics. *PLoS Comput. Biol.* **19**, e1011574 (2023).

52. M. Baiocchi, J. Cheng, D. S. Small, Instrumental variable methods for causal inference. *Stat. Med.* **33**, 2297–2340 (2014).

53. S. Tonegawa, M. D. Morrissey, T. Kitamura, The role of engram cells in the systems consolidation of memory. *Nat. Rev. Neurosci.* **19**, 485–498 (2018).

54. B. F. Grewe, J. Grundemann, L. J. Kitch, J. A. Lecoq, J. G. Parker, J. D. Marshall, M. C. Larkin, P. E. Jercog, F. Grenier, J. Z. Li, A. Luthi, M. J. Schnitzer, Neural ensemble dynamics underlying a long-term associative memory. *Nature* **543**, 670–675 (2017).

55. J. K. Chapin, M. A. Nicolelis, Principal component analysis of neuronal ensemble activity reveals multidimensional somatosensory representations. *J. Neurosci. Methods* **94**, 121–140 (1999).

56. A. Abid, M. J. Zhang, V. K. Bagaria, J. Zou, Exploring patterns enriched in a dataset with contrastive principal component analysis. *Nat. Commun.* **9**, 2134 (2018).

57. Y. Huang, H. Jiang, Q. Zheng, A. H. K. Fok, X. Li, C. G. Lau, C. S. W. Lai, Environmental enrichment or selective activation of parvalbumin-expressing interneurons ameliorates synaptic and behavioral deficits in animal models with schizophrenia-like behaviors during adolescence. *Mol. Psychiatry* **26**, 2533–2552 (2021).

58. M. Sehgal, D. A. Filho, G. Kastellakis, S. Kim, J. Lee, Y. Shen, S. Huang, A. Lavi, G. Fernandes, I. Davila Mejia, S. S. Martin, A. Pekcan, M. S. Wu, W. D. Heo, P. Poirazi, J. T. Trachtenberg, A. J. Silva, Compartmentalized dendritic plasticity in the mouse retrosplenial cortex links contextual memories formed close in time. *Nat. Neurosci.* **28**, 602–615 (2025).

59. A. C. Frank, S. Huang, M. Zhou, A. Gdalyahu, G. Kastellakis, T. K. Silva, E. Lu, X. Wen, P. Poirazi, J. T. Trachtenberg, A. J. Silva, Hotspots of dendritic spine turnover facilitate clustered spine addition and learning and memory. *Nat. Commun.* **9**, 422 (2018).

60. H. Xu, L. Liu, Y. Tian, J. Wang, J. Li, J. Zheng, H. Zhao, M. He, T. L. Xu, S. Duan, H. Xu, A disinhibitory microcircuit mediates conditioned social fear in the prefrontal cortex. *Neuron* **102**, 668–682.e5 (2019).

61. F. W. Grillo, L. West, V. De Paola, Removing synaptic brakes on learning. *Nat. Neurosci.* **18**, 1062–1064 (2015).

62. S. X. Chen, A. N. Kim, A. J. Peters, T. Komiyama, Subtype-specific plasticity of inhibitory circuits in motor cortex during motor learning. *Nat. Neurosci.* **18**, 1109–1115 (2015).

63. C. Scholl, M. E. Rule, M. H. Hennig, The information theory of developmental pruning: Optimizing global network architectures using local synaptic rules. *PLoS Comput. Biol.* **17**, e1009458 (2021).

64. J. P. Weber, B. K. Andrasfalvy, M. Polito, A. Mago, B. B. Ujfalussy, J. K. Makara, Location-dependent synaptic plasticity rules by dendritic spine cooperativity. *Nat. Commun.* **7**, 11380 (2016).

65. T. Branco, B. A. Clark, M. Hausser, Dendritic discrimination of temporal input sequences in cortical neurons. *Science* **329**, 1671–1675 (2010).

66. W. C. Oh, L. K. Parajuli, K. Zito, Heterosynaptic structural plasticity on local dendritic segments of hippocampal CA1 neurons. *Cell Rep.* **10**, 162–169 (2015).

67. Y. Ramiro-Cortes, A. F. Hobbiss, I. Israely, Synaptic competition in structural plasticity and cognitive function. *Philos. Trans. R. Soc. Lond. B Biol. Sci.* **369**, 20130157 (2014).

68. W. C. Oh, T. C. Hill, K. Zito, Synapse-specific and size-dependent mechanisms of spine structural plasticity accompanying synaptic weakening. *Proc. Natl. Acad. Sci. U.S.A.* **110**, E305–E312 (2013).

69. T. E. Chater, M. F. Eggl, Y. Goda, T. Tchumatchenko, Competitive processes shape multi-synapse plasticity along dendritic segments. *Nat. Commun.* **15**, 7572 (2024).

70. G. Kastellakis, S. Tasciotti, I. Pandi, P. Poirazi, The dendritic engram. *Front. Behav. Neurosci.* **17**, 1212139 (2023).

71. J. K. O'Hare, K. C. Gonzalez, S. A. Herrlinger, Y. Hirabayashi, V. L. Hewitt, H. Blockus, M. Szoboszlay, S. V. Rolotti, T. C. Geiller, A. Negrean, V. Chelur, F. Polleux, A. Losonczy, Compartment-specific tuning of dendritic feature selectivity by intracellular Ca^{2+} release. *Science* **375**, eabm1670 (2022).

72. P. J. Dittmer, M. L. Dell'Acqua, W. A. Sather, Synaptic crosstalk conferred by a zone of differentially regulated Ca^{2+} signaling in the dendritic shaft adjoining a potentiated spine. *Proc. Natl. Acad. Sci. U.S.A.* **116**, 13611–13620 (2019).

73. S. M. Short, K. D. Oikonomou, W. L. Zhou, C. D. Acker, M. A. Popovic, D. Zecevic, S. D. Antic, The stochastic nature of action potential backpropagation in apical tuft dendrites. *J. Neurophysiol.* **118**, 1394–1414 (2017).

74. R. L. Clem, D. Schiller, New learning and unlearning: Strangers or accomplices in threat memory attenuation? *Trends Neurosci.* **39**, 340–351 (2016).

75. G. J. Quirk, D. Mueller, Neural mechanisms of extinction learning and retrieval. *Neuropsychopharmacology* **33**, 56–72 (2008).

76. P. Thevenaz, U. E. Ruttimann, M. Unser, A pyramid approach to subpixel registration based on intensity. *IEEE Trans. Image Process.* **7**, 27–41 (1998).

77. M. Tjia, X. Yu, L. S. Jammu, J. Lu, Y. Zuo, Pyramidal neurons in different cortical layers exhibit distinct dynamics and plasticity of apical dendritic spines. *Front. Neural Circuits* **11**, 43 (2017).

78. T. P. Patel, K. Man, B. L. Firestein, D. F. Meaney, Automated quantification of neuronal networks and single-cell calcium dynamics using calcium imaging. *J. Neurosci. Methods* **243**, 26–38 (2015).

79. M. L. Hines, N. T. Carnevale, The NEURON simulation environment. *Neural Comput.* **9**, 1179–1209 (1997).

80. S. Dura-Bernal, B. A. Suter, P. Gleeson, M. Cantarelli, A. Quintana, F. Rodriguez, D. J. Kedziora, G. L. Chadderdon, C. C. Kerr, S. A. Neymotin, R. A. McDougal, M. Hines, G. M. Shepherd, W. W. Lytton, NetPyNE, a tool for data-driven multiscale modeling of brain circuits. *eLife* **8**, e44494 (2019).

81. D. Hasegan, M. Deible, C. Earl, D. D'Onofrio, H. Hazan, H. Anwar, S. A. Neymotin, Training spiking neuronal networks to perform motor control using reinforcement and evolutionary learning. *Front. Comput. Neurosci.* **16**, 1017284 (2022).

82. I. Rabinowitch, I. Segev, The endurance and selectivity of spatial patterns of long-term potentiation/depression in dendrites under homeostatic synaptic plasticity. *J. Neurosci.* **26**, 13474–13484 (2006).

83. Z. Yu, M. Guindani, S. F. Grieco, L. Chen, T. C. Holmes, X. Xu, Beyond *t* test and ANOVA: Applications of mixed-effects models for more rigorous statistical analysis in neuroscience research. *Neuron* **110**, 21–35 (2022).

84. J. Sun, L. Zhu, X. Fang, Y. Tang, Y. Xiao, S. Jiang, J. Lin, Y. Li, Pupil dilation and behavior as complementary measures of fear response in mice. *Cogn. Neurodyn.* **18**, 4047–4054 (2024).
